# Event-Triggered State Filter Estimation for Nonlinear Systems with Packet Dropout and Correlated Noise

**DOI:** 10.3390/s24030769

**Published:** 2024-01-24

**Authors:** Guorui Cheng, Jingang Liu, Shenmin Song

**Affiliations:** Center for Control Theory and Guidance Technology, Harbin Institute of Technology, Harbin 150001, China; 22b904066@stu.hit.edu.cn (J.L.); songshenmin@hit.edu.cn (S.S.)

**Keywords:** correlated noise, cubature Kalman filter, event-triggered mechanism, nonlinear system, packet dropout, performance analysis, state estimation

## Abstract

This paper begins by exploring the challenge of event-triggered state estimations in nonlinear systems, grappling with packet dropout and correlated noise. A communication mechanism is introduced that determines whether to transmit measurement values based on whether event-triggered conditions are violated, thereby minimizing redundant communication data. In designing the filter, noise decorrelation is initially conducted, followed by the integration of the event-triggered mechanism and the unreliable network transmission system for state estimator development. Subsequently, by combining the three-degree spherical–radial cubature rule, the numerical implementation steps of the proposed state estimation framework are outlined. The performance estimation analysis highlights that by adjusting the event-triggered threshold appropriately, the estimation performance and transmission rate can be effectively balanced. It is established that when there is a lower bound on the packet dropout rate, the covariance matrix of the state estimation error remains bounded, and the stochastic stability of the state estimation error is also confirmed. Ultimately, the algorithm and conclusions that are proposed in this paper are validated through a simulation example of a target tracking system.

## 1. Introduction

In recent years, wireless sensor networks (WSNs) have garnered increasing attention and have found widespread applications, particularly in domains like factory manufacturing, target monitoring, and target tracking. A pivotal challenge in wireless sensor networks is system state estimation. Techniques like Kalman filtering (KF) can process signals to refine the data that are transmitted to the estimator, thereby deriving the internal state of the system, which evolves in a consistent manner. However, wireless sensor networks face communication constraints, particularly in bandwidth-limited environments where the energy of each sensor node is limited, making it impractical to transmit voluminous sensor measurement data under such conditions [1,2]. Therefore, it is imperative to adopt strategies that can reduce the communication rate and alleviate the communication burden on the network system while ensuring satisfactory performance in terms of state estimation.

Then, numerous state filters based on an event-triggered mechanism have been introduced for linear systems. Unlike traditional periodic sampling methods, the event-triggered mechanism determines whether to transmit measurement data by assessing whether the event-triggered conditions have been violated. By meticulously selecting appropriate event-triggered mechanisms and thresholds, the communication rates in network systems can be significantly reduced [3]. In [4], an event-triggered mechanism was first integrated into the state estimation process, and it is worth noting that the event-triggered mechanism, along with a time-triggered mechanism, offers commendable estimation accuracy while also achieving lower communication rates.

Inspired by this result, a variety of event-triggered mechanisms have been introduced. For instance, if the difference between the current sensor observation and the previous observation exceeds a predefined threshold, data transmission is triggered [5]. Subsequently, an innovation-based event-triggered mechanism was introduced, which relies on the difference between the current observation value and its predicted value as a metric, rather than the last sent measurement value [6,7]. Some scholars have also proposed a variance-based data transmission mechanism, which only transmits data when the covariance exceeds a specific threshold [8]. Subsequently, reference [9] derived the minimum mean square error (MMSE) filter based on Kalman filtering and innovative conditions. To maintain Gaussian characteristics based on innovation conditions, [10] introduced an event-triggered MMSE filter, grounded in random innovation conditions.

The filters of the event-triggered mechanism designed above are primarily tailored for linear systems, yet in practical engineering applications, ideal linear systems are rare. Therefore, to achieve state estimation in wireless sensor networks (WSN), the introduction of nonlinear filters is essential. There are two well-established methods of state estimation for nonlinear systems. The first is the linearization approach, which includes the extended Kalman filter (EKF) [11] and the divided difference filter (DDF) [12]. However, when the EKF encounters strongly nonlinear models, the state estimation can become unstable. Alternatively, one can employ deterministic sampling methods to compute Gaussian weighted integrals. These methods include the unscented Kalman filter (UKF) [13], which relies on unscented transformation, and the Cubature Kalman filter (CKF) [14], which is based on the spherical–radial cubature rule. There is also the particle filter (PF) [15] algorithm, which relies on random sampling but has the disadvantage of high computational complexity. Notably, it has been demonstrated that the CKF [14] offers better numerical stability, higher accuracy than the UKF, and lower computational complexity than the PF.

The research outcomes detailed above were obtained under conditions where noise was uncorrelated. However, in practical applications, noise correlation can significantly impact the performance of filters. Therefore, numerous scholars have developed state estimators that account for correlated noise in network systems [16,17,18,19,20,21]. Zhao [16] devised a Gaussian filter for nonlinear uncertainty systems with correlated noise and further extended it to handle one-step random delay measurements, packet dropouts, and correlated noise [17]. Tan created a Gaussian recursive filter that accounts for multi-step correlated noise and packet dropout compensation [18]. Sun [19] designed a globally optimal distributed and sequential fusion filter with cross-correlated noise under linear system conditions, which was later extended to incorporate event-triggered conditions for sequential fusion filters [20]. Cheng further extended this to nonlinear systems and implemented it numerically using the CKF method [21].

Given the inevitable presence of nonideal scenarios in network usage, such as network latency and data packet dropout, the design of nonlinear filters with packet dropout has become a pivotal research focus. The stochastic stability of these filters holds immense significance for their performance. In [22], the stochastic stability of extended Kalman filters under packet dropout was examined, revealing that when there is a lower bound on the communication rate, the estimation error remains bounded. Subsequently, [23] delved into the stochastic stability of an improved EKF over fading channels. In [24], a nonlinear system state estimator with an event-triggered mechanism and based on unscented Kalman filtering was introduced, along with its performance analysis under random event-triggered scheduling. According to [25], it is assumed that when dealing with the state estimation problem of nonlinear systems with event-triggered mechanisms, the posterior probability density function of the state may not follow a Gaussian distribution. Nevertheless, as per the above sources and [26], when addressing the state estimation issue in nonlinear systems with event-triggered mechanisms, it is postulated that all conditional probability densities sampled using such mechanisms maintain an approximate Gaussian distribution.

Inspired by the existing literature, there is a paucity of research that simultaneously considers state estimation with event-triggered measurement data transmission mechanisms, noise correlation, and packet dropout. There is no research on using event-triggered mechanisms to design filters for nonlinear systems with correlated noise and packet dropout using traditional methods. The literature that has emerged mainly focuses on the study of linear systems. On this basis, this paper studies the problem of event-triggered state estimation for nonlinear systems with packet dropout and correlated noise, presents the filter design and numerical implementation method, and describes the relationship between the event-triggered threshold and packet dropout rate when the estimation error covariance matrix is bounded. Finally, it is proven that the state estimation error is stochastically stable in the mean square sense.

The main contributions of this paper include (1) that an event-triggered mechanism has been proposed, which can reduce the transmission of communication data and achieve a balance between transmission communication rate and filter estimator performance by adjusting the event-triggered threshold appropriately; (2) on this basis, a new recursive event-triggered state estimator with and packet dropout and correlated noise is proposed for networked nonlinear systems based on the matrix form of a Cubature Kalman filter (CKF); (3) the performance of the state estimator is evaluated, and the relationship between the event-triggered threshold and packet dropout rate is given, ensuring the error convergence and stochastic stability of the filter.

The main difficulty encountered in the research process is how to use the event-triggered mechanism correctly. It is necessary to consider the design process of filters for correlated noise when triggering and not triggering and to conduct comprehensive design at the same time. Secondly, when using CKF for numerical implementation, it is necessary to correctly calculate each variable. Finally, in the estimation performance analysis stage, it is necessary to draw on research [27] that proves the stochastic stability of the CKF for proof.

The sections of this paper are arranged as follows: Section 2 introduces the problem statement and preliminaries; Section 3 presents the design of the event-triggered state estimator with packet dropout and correlated noise; Section 4 presents the numerical implementation steps based on the third-degree spherical–radial cubature rule; Section 5 analyzes the performance of the state estimator; Section 6 uses target tracking simulation examples to verify the algorithm and related conclusions of this paper; and Section 7 summarizes the research in this paper.

Notations: $\delta_{ij}$ denotes the Kronecker Delta function; E[·|·] denotes the conditional expectation; p(x|y) represents the probability distribution of x with respect to y; superscript T and −1 denote the transpose and inverse of matrix; ${\mathbb{R}}^{n}$ represents the n-dimensional Euclidean space; and ℕ denotes the natural number set.

## 2. Problem Statement and Preliminaries

Consider the following nonlinear discrete time model

(1) $$x_{k+1}=f\left(x_{k}\right)+\omega_{k}$$

(2) $$y_{k}=h\left(x_{k}\right)+\upsilon_{k}$$

where $x_{k}\in {\mathbb{R}}^{n}$ is the state vector that needs to be estimated, and $y_{k}\in {\mathbb{R}}^{m}$ is the observation vector; f(·) and h(·) are known nonlinear functions; $\omega_{k}\in {\mathbb{R}}^{n}$ and $\upsilon_{k}\in {\mathbb{R}}^{m}$ are the system noise and observation noise, both satisfying zero-mean Gaussian distributions; and $E\left[w_{k}w_{l}^{T}\right]=Q_{k}\delta_{kl}$, $E\left[v_{k}v_{l}^{T}\right]=R_{k}\delta_{kl}$, and $E\left[w_{k}v_{l}^{T}\right]=S_{k}\delta_{kl}$. The initial state $x_{0}$ obeys Gaussian distribution $\left(x_{0},P_{0}\right)$. In addition, $x_{0}$, $\omega_{k}$, and $\upsilon_{k}$ are independent of each other.

### 2.1. Event-Triggered Mechanism

Due to energy and bandwidth limitations in wireless sensor networks, event-triggered communication strategies are adopted to reduce communication rates.

This article uses a random variable $\gamma_{k}$ to model the above event-triggered process:

(3) $$\gamma_{k}=\left\{ \begin{array}{l} 1,\;if{\left(y_{k}-{\bar{y}}_{k-1}\right)}^{T}\left(y_{k}-{\bar{y}}_{k-1}\right)>\rho \\ 0,\;otherwise \end{array}\right.$$

In the above equation, ${\bar{y}}_{k-1}$ represents the previous measurement data received by the estimator, and ρ represents the event-triggered threshold, which is a positive number. And when $\gamma_{k}=1$, the measurement data $y_{k}$ will be sent to the estimator; otherwise, it will not be sent. Afterwards, the measurement data at time k received by estimator can be modeled as

(4) $${\bar{y}}_{k}=\gamma_{k}y_{k}+\left(1-\gamma_{k}\right){\bar{y}}_{k-1}$$

This means that when $\gamma_{k}=1$, the measurement data point $y_{k}$ is used for state estimation, and if $\gamma_{k}=0$, the previous measurement data point ${\bar{y}}_{k-1}$ is used for state estimation measurement updates.

**Remark** **1.**  *The event-triggered mechanism adopted in (3) does not require information to be returned to the sensor from a remote estimator, which can save communication costs.*

### 2.2. Packet Dropouts

In this article, we assume that the communication link is unreliable, indicating that measurement data may be lost during transmission. The random variable $\lambda_{k}$ is to be defined the phenomenon of packet dropout.

(5) $$\lambda_{k}=\left\{ \begin{array}{l} 1,\;\mathrm{data}\;\mathrm{are}\;\mathrm{received}\\ 0,\;\mathrm{otherwise} \end{array}\right.$$

Note that research has shown that we can define the probability density function of the observation noise under packet dropout conditions:

(6) $$p\left(v_{k}|\lambda_{k}\right)=\left\{ \begin{array}{l} N\left(0,R_{k}\right),\lambda_{k}=1\\ N\left(0,\sigma^{2}I\right),\lambda_{k}=0 \end{array}\right.$$

where σ→∞. And when $\lambda_{k}=1$, it indicates the normal arrival of measurement data $y_{k}$, and when $\lambda_{k}=0$, it indicates the dropout of measurement data $y_{k}$.

The expression of the estimation and estimation error covariance can be expressed as follows:

(7) $$x_{k+1|k}=E\left[x_{k+1}|Y_{k}\right]$$

(8) $$x_{k+1|k+1}=E\left[x_{k+1}|Y_{k+1}\right]$$

(9) $$P_{k+1|k}=E\left[\left(x_{k+1}-x_{k+1|k}\right){\left(x_{k+1}-x_{k+1|k}\right)}^{T}|Y_{k}\right]$$

(10) $$P_{k+1|k+1}=E\left[\left(x_{k+1}-x_{k+1|k+1}\right){\left(x_{k+1}-x_{k+1|k+1}\right)}^{T}|Y_{k+1}\right]$$

where $Y_{k+1}=\left\{\begin{array}{l} \gamma_{0},\gamma_{1},\cdots ,\gamma_{k+1},\lambda_{0},\lambda_{1},\cdots ,\lambda_{k+1},\\ \gamma_{0}\lambda_{0}y_{0},\gamma_{1}\lambda_{1}y_{1},\cdots ,\gamma_{k+1}\lambda_{k+1}y_{k+1} \end{array}\right\}$.

The purpose of this paper is to design an event-triggered state estimator for nonlinear systems with packet dropout and correlated noise.

## 3. The Design of Event-Triggered State Estimation with Packet Dropout and Correlated Noise

In this section, the recursive state estimator of the event-triggered nonlinear system with packet dropouts and correlated noise is derived.

Firstly, it is necessary to decorrelate the process noise and measurement noise:

Let $x_{k+1}=f\left(x_{k}\right)+\omega_{k}+J_{k}\left[y_{k}-h\left(x_{k}\right)-v_{k}\right]$ and $J_{k}=S_{k}R_{k}^{-1}$; then, $x_{k+1}$ can be rewritten as

(11) $$x_{k+1}=F\left(x_{k}\right)+{\bar{\omega }}_{k}$$

where $F\left(x_{k}\right)=f\left(x_{k}\right)+J_{k}\left[y_{k}-h\left(x_{k}\right)\right]$ and ${\bar{\omega }}_{k}=\omega_{k}-S_{k}R_{k}^{-1}v_{k}$. So, we have that

(12) $$E\left[{\bar{\omega }}_{k}{\bar{\omega }}_{l}^{T}\right]=Q_{k}^{*}\delta_{kl}=\left(Q_{k}-S_{k}R_{k}^{-1}S_{k}^{T}\right)\delta_{kl}$$

(13) $$E\left[{\bar{\omega }}_{k}\upsilon_{k}^{T}\right]=0$$

Therefore, the design of the filter estimator can be carried out using equivalent systems (11) and (2) after noise decorrelation. Before designing the filter, Lemma 1 needs to be introduced.

**Lemma** **1.**  *For any two vectors* where $a,b\in {\mathbb{R}}^{n}$*,* ε>0*, and* ε *is a scalar, the following inequality holds:*

(14) $$ab^{T}+ba^{T}\leq \varepsilon aa^{T}+\varepsilon^{-1}bb^{T}$$

**Theorem** **1.**  *Combining the numerical realization method of the nonlinear system with the matrix expression [27], the recursive state estimator of the event-triggered nonlinear system (11) and (2) with packet dropouts and correlated noise can be obtained as follows:*

*Time Update:*

(15) $$\begin{array}{cc} {\hat{x}}_{k+1|k} & =\int f\left(x_{k}\right)N\left(x_{k};{\hat{x}}_{k|k},{\hat{P}}_{k|k}\right)dx_{k}\\ & +\gamma_{k}\lambda_{k}J_{k}\left[y_{k}-\int h\left(x_{k}\right)N\left(x_{k};{\hat{x}}_{k|k},{\hat{P}}_{k|k}\right)dx_{k}\right] \end{array}$$

(16) $$P_{k+1|k}=A_{k}{\bar{P}}_{k|k}A_{k}^{T}+Q_{k}+\gamma_{k}\lambda_{k}\left(J_{k}C_{k}{\bar{P}}_{k|k}C_{k}^{T}J_{k}^{T}-J_{k}R_{k}J_{k}^{T}-J_{k}C_{k}{\bar{P}}_{k|k}A_{k}^{T}-A_{k}{\bar{P}}_{k|k}C_{k}^{T}J_{k}^{T}\right)$$

*Measurement Update:*

(17) $$\begin{array}{l} {\hat{x}}_{k+1}={\hat{x}}_{k+1|k}+\gamma_{k+1}K_{k+1}\left(y_{k+1}-{\hat{y}}_{k+1}\right)\\ +\left(1-\gamma_{k+1}\right)L_{k+1}\left({\bar{y}}_{k+1}-{\hat{y}}_{k+1|k}\right) \end{array}$$

(18) $$K_{k+1}=P_{k+1|k}B_{k+1}^{T}{\left(\begin{array}{l} B_{k+1}P_{k+1|k}B_{k+1}^{T}\\ +\lambda_{k+1}R_{k+1}+\left(1-\lambda_{k+1}\right)\sigma^{2}I \end{array}\right)}^{-1}$$

(19) $$\begin{array}{l} L_{k+1}=\left(1+a_{1}\right)P_{k+1|k}B_{k+1}^{T}\\ \times {\left[\begin{array}{l} \left(1+a_{1}\right)B_{k+1}P_{k+1|k}B_{k+1}^{T}\\ +\left(1+a_{2}\right)R_{k+1}+\left(1+a_{1}^{-1}+a_{2}^{-1}\right)\rho I \end{array}\right]}^{-1} \end{array}$$

(20) $$\begin{array}{l} {\bar{P}}_{k+1}=P_{k+1|k}-\gamma_{k+1}K_{k+1}B_{k+1}P_{k+1|k}+\left(1-\gamma_{k+1}\right)\\ \times \left[\begin{array}{l} \left(1+a_{1}\right)\left(I-L_{k+1}B_{k+1}\right)P_{k+1|k}{\left(I-L_{k+1}B_{k+1}\right)}^{T}\\ +\left(1+a_{2}\right)L_{k+1}R_{k+1}L_{k+1}^{T}\\ +\left(1+a_{1}^{-1}+a_{2}^{-1}\right)L_{k+1}\rho IL_{k+1}^{T}-P_{k+1|k} \end{array}\right] \end{array}$$

*where *${\hat{y}}_{k+1|k}=B_{k+1}{\hat{x}}_{k+1|k}$*, *${\bar{y}}_{k}=\gamma_{k}y_{k}+\left(1-\gamma_{k}\right){\bar{y}}_{k-1}$*, and *σ→∞* when *$\lambda_{k+1}=0$*. And *${\bar{P}}_{k+1}$* is the upper bound of *$P_{k+1|k+1}$* in * (10).

(21) $$P_{k+1|k+1}\leq {\bar{P}}_{k+1}$$

**Proof.**  First, on the basis of the event-triggered state estimation and then considering reliable and unreliable networks, the proof is divided into two parts.

Step 1: Consider transmitting in a reliable network, which only considers an event-triggered mechanism and does not consider packet dropout. Let ${\overset{⌣}{Y}}_{k}=\left\{\gamma_{0},\gamma_{1},\cdots ,\gamma_{k},\gamma_{0}y_{0},\gamma_{11}y_{1},\cdots ,\gamma_{k}y_{k}\right\}$ and define ${\overset{⌣}{x}}_{k|k}≜\left\{{\overset{⌣}{x}}_{k}|{\overset{⌣}{Y}}_{k}\right\}$, ${\overset{⌣}{x}}_{k|k-1}≜\left\{{\overset{⌣}{x}}_{k}|{\overset{⌣}{Y}}_{k-1}\right\}$, ${\overset{⌣}{P}}_{k|k}≜\left\{\left(x_{k}-{\overset{⌣}{x}}_{k|k}\right){\left(x_{k}-{\overset{⌣}{x}}_{k|k}\right)}^{T}|{\overset{⌣}{Y}}_{k}\right\}$, and ${\overset{⌣}{P}}_{k|k-1}≜\left\{\left(x_{k}-{\overset{⌣}{x}}_{k|k-1}\right){\left(x_{k}-{\overset{⌣}{x}}_{k|k-1}\right)}^{T}|{\overset{⌣}{Y}}_{k}\right\}$.

Time Update:

If $\gamma_{k}=1$, this indicates that the measurement information $y_{k}$ at time k has arrived normally, and the process noise $w_{k}$ and measurement noise $v_{k}$ are correlated. Therefore, it is necessary to use a decorrelation system (11) for calculation.

(22) $$\begin{array}{cc} {\overset{⌣}{x}}_{k+1|k} & =E\left[f\left(x_{k}\right)+J_{k}\left[y_{k}-h\left(x_{k}\right)\right]|Y_{k}\right]\\ & =E\left[f\left(x_{k}\right)|Y_{k}\right]+J_{k}\left[y_{k}-h\left(x_{k}\right)|Y_{k}\right]\\ & =\int f\left(x_{k}\right)N\left(x_{k};{\hat{x}}_{k|k},{\hat{P}}_{k|k}\right)dx_{k}\\ & +J_{k}\left[y_{k}-\int h\left(x_{k}\right)N\left(x_{k};{\hat{x}}_{k|k},{\hat{P}}_{k|k}\right)dx_{k}\right] \end{array}$$

By using the cubature rule [14], it can be obtained that

(23) $${\overset{⌣}{x}}_{k+1|k}=\frac{1}{2n_{x}}\sum_{i=1}^{2n_{x}}X_{i,k+1|k}^{*}+J_{k}\left[y_{k}-\frac{1}{2n_{x}}\sum_{i=1}^{2n_{x}}{\bar{X}}_{i,k+1|k}^{*}\right]$$

The expression for the prediction error of the state estimation is as follows:

(24) $${\tilde{x}}_{k+1|k}=x_{k+1}-{\overset{⌣}{x}}_{k+1|k}$$

Perform Taylor expansion on $f\left(x_{k}\right)$, $h\left(x_{k}\right)$ and substitute (23) into (24) to obtain $x_{k+1|k}=\left(\nabla f\left({\hat{x}}_{k|k}\right)-J_{k}\nabla h\left({\hat{x}}_{k|k}\right)\right){\tilde{x}}_{k|k}+{\bar{w}}_{k}$, where $\nabla =\frac{\partial }{\partial x}|x={\hat{x}}_{k|k}$. The above equation can be converted to

(25) $${\tilde{x}}_{k+1|k}=\left(A_{k}-J_{k}C_{k}\right){\tilde{x}}_{k|k}+{\bar{\omega }}_{k}$$

where $A_{k}=\alpha_{k}F_{k}$, $F_{k}=\frac{\partial f\left(x\right)}{\partial x}|x={\hat{x}}_{k|k}$, $C_{k}={\tilde{\alpha }}_{k}{\tilde{C}}_{k}$, ${\tilde{C}}_{k}=\frac{\partial h\left(x\right)}{\partial x}|x={\hat{x}}_{k|k}$ is the Jacobian matrix and $\alpha_{k}=diag\left(\alpha_{1,k},\alpha_{2,k},\cdots ,\alpha_{n_{x},k}\right)$, and ${\tilde{\alpha }}_{k}=diag\left({\tilde{\alpha }}_{1,k},{\tilde{\alpha }}_{2,k},\cdots ,{\tilde{\alpha }}_{n_{x},k}\right)$ is an unknown diagonal matrix representing the higher-order terms. And let $A_{k}^{*}=A_{k}-J_{k}C_{k}$.

And the covariance matrix of the state prediction error is

(26) $${\overset{⌣}{P}}_{k+1|k}=E\left[{\tilde{x}}_{k+1|k}{\tilde{x}}_{k+1|k}^{T}\right]=A_{k}^{*}{\overset{⌣}{P}}_{k|k}{\left(A_{k}^{*}\right)}^{T}+Q_{k}^{*}$$

When $\gamma_{k}=0$, it indicates that the measurement information $y_{k}$ at time k has not arrived normally. Therefore, it is necessary to use system (1) without correlated noise and then proceed with the steps of time update.

(27) $$\begin{array}{cc} {\overset{⌣}{x}}_{k+1|k} & =E\left[f\left(x_{k}\right)+w_{k}|Y_{k}\right]\\ & =\int f\left(x_{k}\right)N\left(x_{k};{\hat{x}}_{k|k},{\hat{P}}_{k|k}\right)dx_{k} \end{array}$$

(28) $$\begin{array}{cc} {\tilde{x}}_{k+1|k} & =x_{k+1}-{\overset{⌣}{x}}_{k+1|k}\\ & =f\left(x_{k}\right)+w_{k}-\int f\left(x_{k}\right)N\left(x_{k};{\hat{x}}_{k|k},{\hat{P}}_{k|k}\right)dx_{k}\\ & =A_{k}{\tilde{x}}_{k|k}+\omega_{k} \end{array}$$

(29) $${\overset{⌣}{P}}_{k+1|k}=A_{k}{\overset{⌣}{P}}_{k|k}A_{k}^{T}+Q_{k}$$

Combining Equations (22)–(29), it can be found that

(30) $$\begin{array}{cc} {\overset{⌣}{x}}_{k+1|k} & =\int f\left(x_{k}\right)N\left(x_{k};{\hat{x}}_{k|k},{\hat{P}}_{k|k}\right)dx_{k}\\ & +\gamma_{k}J_{k}\left[y_{k}-\int h\left(x_{k}\right)N\left(x_{k};{\hat{x}}_{k|k},{\hat{P}}_{k|k}\right)dx_{k}\right] \end{array}$$

(31) $${\overset{⌣}{P}}_{k+1|k}=A_{k}{\overset{⌣}{P}}_{k|k}A_{k}^{T}+Q_{k}+\gamma_{k}\left(J_{k}C_{k}{\overset{⌣}{P}}_{k|k}C_{k}^{T}J_{k}^{T}-J_{k}R_{k}J_{k}^{T}-J_{k}C_{k}{\overset{⌣}{P}}_{k|k}A_{k}^{T}-A_{k}{\overset{⌣}{P}}_{k|k}C_{k}^{T}J_{k}^{T}\right)$$

Measurement Update:

When $\gamma_{k+1}=1$, the measurement data point $y_{k+1}$ arrives normally, as it is a normal measurement update process.

(32) $${\overset{⌣}{x}}_{k+1|k+1}={\overset{⌣}{x}}_{k+1|k}+{\overset{⌣}{K}}_{k+1|k+1}\left(y_{k}-{\overset{⌣}{y}}_{k+1|k}\right)$$

(33) $${\overset{⌣}{y}}_{k+1|k}=B_{k+1}{\overset{⌣}{x}}_{k+1|k}$$

(34) $${\overset{⌣}{P}}_{k+1|k+1}={\overset{⌣}{P}}_{k+1|k}-{\overset{⌣}{P}}_{k+1|k}B_{k+1}^{T}{\left(B_{k+1}{\overset{⌣}{P}}_{k+1|k}B_{k+1}^{T}+R_{k+1}\right)}^{-1}B_{k+1}{\overset{⌣}{P}}_{k+1|k}$$

(35) $${\overset{⌣}{K}}_{k+1|k+1}={\overset{⌣}{P}}_{k+1|k}B_{k+1}^{T}{\left(B_{k+1}{\overset{⌣}{P}}_{k+1|k}B_{k+1}+R_{k+1}\right)}^{-1}$$

When $\gamma_{k+1}=0$, the measurement data point $y_{k+1}$ does not arrives, and the process of measurement update is as follows:

(36) $${\overset{⌣}{x}}_{k+1|k+1}={\overset{⌣}{x}}_{k+1|k}+{\overset{⌣}{L}}_{k+1}\left({\bar{y}}_{k+1}-{\overset{⌣}{y}}_{k+1|k}\right)$$

The above equation can be rewritten as follows:

(37) $${\overset{⌣}{x}}_{k+1}={\overset{⌣}{x}}_{k+1|k}+{\overset{⌣}{L}}_{k+1}\left({\tilde{y}}_{k+1|k}-e_{k+1}\right)$$

where $e_{k+1}=y_{k+1}-{\bar{y}}_{k+1}$.

The state estimation error can be expressed as

(38) $${\overset{⌣}{x}}_{k+1}=\left(I-{\overset{⌣}{L}}_{k+1}B_{k+1}\right){\overset{⌣}{x}}_{k+1|k}-{\overset{⌣}{L}}_{k+1}\upsilon_{k+1}+{\overset{⌣}{L}}_{k+1}e_{k+1}$$

Then, the estimation error covariance matrix can be obtained:

(39) $$\begin{array}{cc} {\overset{⌣}{P}}_{k+1} & =E\left[{\overset{⌣}{x}}_{k+1}{\overset{⌣}{x}}_{k+1}^{T}\right]\\ & =\left(I-{\overset{⌣}{L}}_{k+1}B_{k+1}\right){\overset{⌣}{P}}_{k+1|k}{\left(I-{\overset{⌣}{L}}_{k+1}B_{k+1}\right)}^{T}\\ & +{\overset{⌣}{L}}_{k+1}R_{k+1}{\left({\overset{⌣}{L}}_{k+1}\right)}^{T}+{\overset{⌣}{L}}_{k+1}E\left[e_{k+1}e_{k+1}^{T}\right]{\overset{⌣}{L}}_{k+1}^{T}\\ & +E\left[\begin{array}{l} \left(I-{\overset{⌣}{L}}_{k+1}B_{k+1}\right){\overset{⌣}{x}}_{k+1|k}e_{k+1}^{T}{\overset{⌣}{L}}_{k+1}^{T}\\ +{\overset{⌣}{L}}_{k+1}e_{k+1}{\overset{⌣}{x}}_{k+1|k}^{T}{\left(I-{\overset{⌣}{L}}_{k+1}B_{k+1}\right)}^{T}\\ -{\overset{⌣}{L}}_{k+1}\upsilon_{k+1}e_{k+1}^{T}{\overset{⌣}{L}}_{k+1}^{T}\\ -{\overset{⌣}{L}}_{k+1}e_{k+1}\upsilon_{k+1}^{T}{\overset{⌣}{L}}_{k+1}^{T} \end{array}\right] \end{array}$$

Based on the event-triggered mechanism (3) and Lemma 1, we can obtain the following inequality:

(40) $$\begin{array}{l} \left(I-{\overset{⌣}{L}}_{k+1}B_{k+1}\right){\overset{⌣}{x}}_{k+1|k}e_{k+1}^{T}L_{k+1}^{T}\\ +L_{k+1}e_{k+1}{\overset{⌣}{x}}_{k+1|k}^{T}\left(I-{\overset{⌣}{L}}_{k+1}B_{k+1}\right)\\ \leq a_{1}\left(I-{\overset{⌣}{L}}_{k+1}B_{k+1}\right){\overset{⌣}{x}}_{k+1|k}{\overset{⌣}{x}}_{k+1|k}^{T}{\left(I-{\overset{⌣}{L}}_{k+1}B_{k+1}\right)}^{T}\\ +a_{1}^{-1}{\overset{⌣}{L}}_{k+1}e_{k+1}e_{k+1}^{T}{\overset{⌣}{L}}_{k+1}^{T} \end{array}$$

and

(41) $$\begin{array}{l} -{\overset{⌣}{L}}_{k+1}\upsilon_{k+1}e_{k+1}^{T}{\overset{⌣}{L}}_{k+1}^{T}-{\overset{⌣}{L}}_{k+1}e_{k+1}\upsilon_{k+1}^{T}{\overset{⌣}{L}}_{k+1}^{T}\\ \leq a_{2}{\overset{⌣}{L}}_{k+1}\upsilon_{k+1}\upsilon_{k+1}^{T}{\overset{⌣}{L}}_{k+1}^{T}+a_{2}^{-1}{\overset{⌣}{L}}_{k+1}e_{k+1}e_{k+1}^{T}{\overset{⌣}{L}}_{k+1}^{T} \end{array}$$

where $a_{1}$ and $a_{2}$ are positive scalars.

Substituting Equations (40) and (41) into (39) can obtain the upper bound of the state estimation error covariance matrix.

(42) $$\begin{array}{cc} {\bar{\mathbb{P}}}_{k+1|k+1} & =\left(1+a_{1}\right)\left(I-{\overset{⌣}{L}}_{k+1}B_{k+1}\right){\overset{⌣}{P}}_{k+1|k}{\left(I-{\overset{⌣}{L}}_{k+1}B_{k+1}\right)}^{T}\\ & +\left(1+a_{2}\right){\overset{⌣}{L}}_{k+1}R_{k+1}{\overset{⌣}{L}}_{k+1}^{T}\\ & +\left(1+a_{1}^{-1}+a_{2}^{-1}\right){\overset{⌣}{L}}_{k+1}\rho I{\overset{⌣}{L}}_{k+1}^{T} \end{array}$$

The filter estimation gain ${\overset{⌣}{L}}_{k+1}$ can be calculated using the following equation:

(43) $$\frac{\partial tr\left({\bar{\mathbb{P}}}_{k+1|k+1}\right)}{{\overset{⌣}{L}}_{k+1}}=0$$

So that

(44) $$\begin{array}{l} {\overset{⌣}{L}}_{k+1}=\left(1+a_{1}\right){\overset{⌣}{P}}_{k+1|k}B_{k+1}^{T}\\ \times {\left[\begin{array}{l} \left(1+a_{1}\right)B_{k+1}{\overset{⌣}{P}}_{k+1|k}B_{k+1}^{T}\\ +\left(1+a_{2}\right)R_{k+1}+\left(1+a_{1}^{-1}+a_{2}^{-1}\right)\rho I \end{array}\right]}^{-1} \end{array}$$

In summary, combining (22)–(24) in a reliable network environment, we can derive an event-triggered state estimator for a nonlinear system with correlated noise.

(45) $$\begin{array}{cc} {\overset{⌣}{x}}_{k+1|k} & =\int f\left(x_{k}\right)N\left(x_{k};{\hat{x}}_{k|k},{\hat{P}}_{k|k}\right)dx_{k}\\ & +\gamma_{k}J_{k}\left[y_{k}-\int h\left(x_{k}\right)N\left(x_{k};{\hat{x}}_{k|k},{\hat{P}}_{k|k}\right)dx_{k}\right] \end{array}$$

(46) $${\overset{⌣}{P}}_{k+1|k}=A_{k}{\bar{\mathbb{P}}}_{k|k}A_{k}^{T}+Q_{k}+\gamma_{k}\left(J_{k}C_{k}{\bar{\mathbb{P}}}_{k|k}C_{k}^{T}J_{k}^{T}-J_{k}R_{k}J_{k}^{T}-J_{k}C_{k}{\bar{\mathbb{P}}}_{k|k}A_{k}^{T}-A_{k}{\bar{\mathbb{P}}}_{k|k}C_{k}^{T}J_{k}^{T}\right)$$

(47) $$\begin{array}{l} {\overset{⌣}{x}}_{k+1}={\overset{⌣}{x}}_{k+1|k}+\gamma_{k+1}{\overset{⌣}{K}}_{k+1|k+1}\left(y_{k+1}-{\overset{⌣}{y}}_{k+1|k}\right)\\ +\left(1-\gamma_{k+1}\right){\overset{⌣}{L}}_{k+1}\left({\bar{y}}_{k+1}-{\overset{⌣}{y}}_{k+1|k}\right) \end{array}$$

(48) $${\overset{⌣}{K}}_{k+1|k+1}={\overset{⌣}{P}}_{k+1|k}B_{k+1}^{T}{\left(B_{k+1}{\overset{⌣}{P}}_{k+1|k}B_{k+1}^{T}+R_{k}\right)}^{-1}$$

(49) $$\begin{array}{l} {\overset{⌣}{L}}_{k+1}=\left(1+a_{1}\right){\overset{⌣}{P}}_{k+1|k}B_{k+1}^{T}\\ \times {\left[\begin{array}{l} \left(1+a_{1}\right)B_{k+1}{\overset{⌣}{P}}_{k+1|k}B_{k+1}^{T}\\ +\left(1+a_{2}\right)R_{k+1}+\left(1+a_{1}^{-1}+a_{2}^{-1}\right)\rho I \end{array}\right]}^{-1} \end{array}$$

(50) $$\begin{array}{l} {\bar{\mathbb{P}}}_{k+1}={\overset{⌣}{P}}_{k+1|k}-\gamma_{k+1}{\overset{⌣}{K}}_{k+1}B_{k+1}{\overset{⌣}{P}}_{k+1|k}+\left(1-\gamma_{k+1}\right)\\ \times \left[\begin{array}{l} \left(1+a_{1}\right)\left(I-{\overset{⌣}{L}}_{k+1}B_{k+1}\right){\overset{⌣}{P}}_{k+1|k}{\left(I-{\overset{⌣}{L}}_{k+1}B_{k+1}\right)}^{T}\\ +\left(1+a_{2}\right){\overset{⌣}{L}}_{k+1}R_{k+1}{\overset{⌣}{L}}_{k+1}^{T}\\ +\left(1+a_{1}^{-1}+a_{2}^{-1}\right){\overset{⌣}{L}}_{k+1}\rho I{\overset{⌣}{L}}_{k+1}^{T}-{\overset{⌣}{P}}_{k+1|k} \end{array}\right] \end{array}$$

Step 2: Consider event-triggered mechanism state estimation with an unreliable network. It should be noted that packet dropout occurs when the estimator responds to the event-triggered mechanism, i.e., $\gamma_{k}=1$. Then, based on [28], a state filter with packet dropout is directly provided.

(51) $$\begin{array}{cc} {\hat{x}}_{k+1|k} & =\int f\left(x_{k}\right)N\left(x_{k};{\hat{x}}_{k|k},{\hat{P}}_{k|k}\right)dx_{k}\\ & +\gamma_{k}\lambda_{k}J_{k}\left[y_{k}-\int h\left(x_{k}\right)N\left(x_{k};{\hat{x}}_{k|k},{\hat{P}}_{k|k}\right)dx_{k}\right] \end{array}$$

(52) $$P_{k+1|k}=A_{k}{\bar{P}}_{k|k}A_{k}^{T}+Q_{k}+\gamma_{k}\lambda_{k}\left(J_{k}C_{k}{\bar{P}}_{k|k}C_{k}^{T}J_{k}^{T}-J_{k}R_{k}J_{k}^{T}-J_{k}C_{k}{\bar{P}}_{k|k}A_{k}^{T}-A_{k}{\bar{P}}_{k|k}C_{k}^{T}J_{k}^{T}\right)$$

(53) $$\begin{array}{l} {\hat{x}}_{k+1}={\hat{x}}_{k+1|k}+\gamma_{k+1}K_{k+1}\left(y_{k+1}-{\hat{y}}_{k+1}\right)\\ +\left(1-\gamma_{k+1}\right)L_{k+1}\left({\bar{y}}_{k+1}-{\hat{y}}_{k+1|k}\right) \end{array}$$

(54) $$K_{k+1}=P_{k+1|k}B_{k+1}^{T}{\left(\begin{array}{l} B_{k+1}P_{k+1|k}B_{k+1}^{T}\\ +\lambda_{k+1}R_{k+1}+\left(1-\lambda_{k+1}\right)\sigma^{2}I \end{array}\right)}^{-1}$$

(55) $$\begin{array}{l} L_{k+1}=\left(1+a_{1}\right)P_{k+1|k}B_{k+1}^{T}\\ \times {\left[\begin{array}{l} \left(1+a_{1}\right)B_{k+1}P_{k+1|k}B_{k+1}^{T}\\ +\left(1+a_{2}\right)R_{k+1}+\left(1+a_{1}^{-1}+a_{2}^{-1}\right)\rho I \end{array}\right]}^{-1} \end{array}$$

(56) $$\begin{array}{l} {\bar{P}}_{k+1}=P_{k+1|k}-\gamma_{k+1}K_{k+1}B_{k+1}P_{k+1|k}+\left(1-\gamma_{k+1}\right)\\ \times \left[\begin{array}{l} \left(1+a_{1}\right)\left(I-L_{k+1}B_{k+1}\right)P_{k+1|k}{\left(I-L_{k+1}B_{k+1}\right)}^{T}\\ +\left(1+a_{2}\right)L_{k+1}R_{k+1}L_{k+1}^{T}\\ +\left(1+a_{1}^{-1}+a_{2}^{-1}\right)L_{k+1}\rho IL_{k+1}^{T}-P_{k+1|k} \end{array}\right] \end{array}$$

where ${\hat{y}}_{k+1|k}=B_{k+1}{\hat{x}}_{k+1|k}$, ${\bar{y}}_{k}=\gamma_{k}y_{k}+\left(1-\gamma_{k}\right){\bar{y}}_{k-1}$, and σ→∞ when $\lambda_{k+1}=0$. □

**Remark** **2.** *The event-triggered threshold* ρ *can be set by specific physical systems and estimation accuracy requirements.* $a_{1}$ *and* $a_{2}$ *are given constants, and the upper bound of* ${\bar{P}}_{k+1}$ *can be adjusted.*

**Remark** **3.**  *This article investigates the state estimation problem of nonlinear systems with correlated noise and packet loss based on an event-triggered mechanism. From (56), it can be seen that the upper bound *${\bar{P}}_{k+1}$* of the state estimation error can be adjusted by adjusting the event-triggering threshold *ρ. * This demonstrates that the design of the event-triggering mechanism and filtering state estimator can achieve a compromise between the transmission rate and estimation performance.*

**Remark** **4.** *The use of this event-triggered mechanism in calculating the gain matrix during filter design has a certain degree of ingenuity, and the state filter estimator uses this event-triggered mechanism to calculate two gain matrices and innovatively adopts the matrix form of the nonlinear system state estimation’s expression.*

**Remark** **5.**  *According to reference [29], the conditional probability density of variables based on the event-triggered mechanism no longer follows a Gaussian distribution, but using non-Gaussian density functions for numerical calculations will become very large. Meanwhile, [30] shows through numerical examples that the conditional distribution can be approximated as a Gaussian distribution, and the approximation error is acceptable. Therefore, in this article, we assume that all variable probability density functions follow a Gaussian distribution.*

## 4. Numerical Implementation Based on Spherical–Radial Cubature Rule

In this section, the numerical implementation steps of the proposed algorithm are provided based on the third-degree spherical–radial cubature rule [14]. And it avoids calculating the Jacobian matrix.

Time Update:

Factorize

(57) $${\hat{P}}_{k|k}=S_{k}S_{k}^{T}$$

Evaluate the cubature points:

(58) $$X_{i,k|k}=S_{k}\xi_{i}+{\hat{x}}_{k|k}$$

Evaluate the propagated cubature points:

(59) $$X_{i,k+1|k}^{*}=f\left(X_{i,k|k}\right)$$

(60) $${\bar{X}}_{i,k+1|k}^{*}=h\left(X_{i,k|k}\right)$$

Estimate the predicted state:

(61) $${\hat{x}}_{k+1|k}=\frac{1}{2n_{x}}\sum_{i=1}^{2n_{x}}X_{i,k+1|k}^{*}+\gamma_{k}\lambda_{k}J_{k}\left[y_{k}-\frac{1}{2n_{x}}\sum_{i=1}^{2n_{x}}{\bar{X}}_{i,k+1|k}^{*}\right]$$

Estimate the predicted error covariance:

(62) $$P_{k+1|k}=A_{k}{\bar{P}}_{k|k}A_{k}^{T}+Q_{k}+\gamma_{k}\lambda_{k}\left(J_{k}C_{k}{\bar{P}}_{k|k}C_{k}^{T}J_{k}^{T}-J_{k}R_{k}J_{k}^{T}-J_{k}C_{k}{\bar{P}}_{k|k}A_{k}^{T}-A_{k}{\bar{P}}_{k|k}C_{k}^{T}J_{k}^{T}\right)$$

Measurement Update:

Factorize

(63) $$P_{k+1|k}=S_{k+1|k}S_{k+1|k}^{T}$$

Evaluate the cubature points:

(64) $$X_{i,k+1|k}=S_{k+1}\xi_{i}+{\hat{x}}_{k+1|k}$$

Evaluate the propagated cubature points:

(65) $$Y_{i,k+1|k}=h\left(X_{i,k+1|k}\right)$$

Estimate the predicted measurement:

(66) $${\hat{y}}_{k+1|k}=\frac{1}{2n_{x}}\sum_{i=1}^{2n_{x}}Y_{i,k+1|k}$$

Estimate the cross-covariance matrix:

(67) $$P_{k+1|k}^{xy}=\frac{1}{2n_{x}}\sum_{i=1}^{2n_{x}}X_{i,k+1|k}Y_{i,k+1|k}^{T}-{\hat{x}}_{k+1|k}{\hat{y}}_{k+1|k}^{T}$$

Estimate the innovation covariance matrix:

(68) $$\begin{array}{l} P_{k+1|k}^{yy}=\frac{1}{2n_{x}}\sum_{i=1}^{2n_{x}}Y_{i,k+1|k}Y_{i,k+1|k}^{T}-{\hat{y}}_{k+1|k}{\hat{y}}_{k+1|k}^{T}\\ +\lambda_{k+1}R_{k+1}+\left(1-\lambda_{k+1}\right)\sigma^{2}I \end{array}$$

(69) $$\begin{array}{ll} {\bar{P}}_{k+1|k}^{\bar{y}\bar{y}} & =\left(1+a_{1}\right)\left(\frac{1}{2n_{x}}\sum_{i=1}^{2n_{x}}Y_{i,k+1|k}Y_{i,k+1|k}^{T}-{\hat{y}}_{k+1|k}{\hat{y}}_{k+1|k}^{T}\right)\\ & +\left(1+a_{2}\right)R_{k+1}+\left(1+a_{1}^{-1}+a_{2}^{-1}\right)\rho I \end{array}$$

Estimate the filter gain:

When $\gamma_{k+1}=1$:

(70) $$K_{k+1}=P_{k+1|k}^{xy}{\left(P_{k+1|k}^{yy}\right)}^{-1}$$

when $\gamma_{k+1}=0$:

(71) $$L_{k+1}=\left(1+a_{1}\right)P_{k+1|k}^{xy}{\left({\bar{P}}_{k+1|k}^{\bar{y}\bar{y}}\right)}^{-1}$$

Estimate the updated state:

(72) $$\begin{array}{l} {\hat{x}}_{k+1}={\hat{x}}_{k+1|k}+\gamma_{k+1}K_{k+1}\left(y_{k+1}-{\hat{y}}_{k+1|k}\right)\\ +\left(1-\gamma_{k+1}\right)L_{k+1}\left({\bar{y}}_{k+1}-{\hat{y}}_{k+1|k}\right) \end{array}$$

Estimate the upper bound of the state estimation error covariance:

(73) $$\begin{array}{l} {\bar{P}}_{k+1}=P_{k+1|k}-\gamma_{k+1}K_{k+1}{\left(P_{k+1|k}^{xy}\right)}^{T}+\left(1-\gamma_{k+1}\right)\\ \times \left[\begin{array}{l} \left(1+a_{1}\right)\left(\begin{array}{l} P_{k+1|k}-P_{k+1|k}^{xy}L_{k+1}^{T}-L_{k+1}{\left(P_{k+1|k}^{xy}\right)}^{T}\\ +L_{k+1}\left(\begin{array}{l} \frac{1}{2n_{x}}\sum_{i=1}^{2n_{x}}Y_{i,k+1|k}Y_{i,k+1|k}^{T}\\ -{\hat{y}}_{k+1|k}{\hat{y}}_{k+1|k}^{T} \end{array}\right)L_{k+1}^{T} \end{array}\right)\\ +\left(1+a_{2}\right)L_{k+1}R_{k+1}L_{k+1}^{T}\\ +\left(1+a_{1}^{-1}+a_{2}^{-1}\right)L_{k+1}\rho IL_{k+1}^{T}\\ -P_{k+1|k} \end{array}\right] \end{array}$$

## 5. Performance Analysis

This section evaluates the performance of the state estimator and provides the relationship between the communication rate and estimation performance.

According to [27,31], referring to the proof that the CKF and EKF have stochastic stability and assuming that the linearized model of systems (11) and (2) is uniformly observable, inequality (74) can be derived:

(74) $$\begin{array}{l} q_{\min}I\leq {\hat{Q}}_{k};Q_{k}\leq q_{\max}I;f_{\min}^{2}I\leq F_{k}F_{k}^{T}\leq f_{\max}^{2}I\\ r_{\min}I\leq R_{k}\leq r_{\max}I;\alpha_{\min}^{2}f_{\min}^{2}I\leq A_{k}A_{k}^{T}\leq \alpha_{\max}^{2}f_{\max}^{2}I\\ \beta_{\min}^{2}I\leq \beta_{k}\beta_{k}^{T}\leq \beta_{\max}^{2}I;\beta_{\min}^{2}h_{\min}^{2}I\leq B_{k}B_{k}^{T}\leq \beta_{\max}^{2}h_{\max}^{2}I\\ h_{\min}^{2}I\leq H_{k}H_{k}^{T}\leq h_{\max}^{2}I;\alpha_{\min}^{2}I\leq \alpha_{k}\alpha_{k}^{T}\leq \alpha_{\max}^{2}I\\ c_{\min}^{2}I\leq {\tilde{C}}_{k}{\tilde{C}}_{k}^{T}\leq c_{\max}^{2}I;{\tilde{\alpha }}_{\min}^{2}I\leq {\tilde{\alpha }}_{k}{\tilde{\alpha }}_{k}^{T}\leq {\tilde{\alpha }}_{\max}^{2}I\\ {\tilde{\alpha }}_{\min}^{2}c_{\min}^{2}I\leq C_{k}C_{k}^{T}\leq {\tilde{\alpha }}_{\max}^{2}c_{\max}^{2}I\\ j_{\min}I\leq J_{k}\leq j_{\max}I;s_{\min}I\leq S_{k}\leq s_{\max}I; \end{array}$$

where $f_{\min},f_{\max},h_{\min},h_{\max},{\tilde{\alpha }}_{\min},{\tilde{\alpha }}_{\max}\alpha_{\min},\alpha_{\max},\beta_{\min},\beta_{\max}\neq 0$, and $r_{\max},q_{\max},{\hat{q}}_{\min},{\hat{r}}_{\min}>0$ are all real numbers.

### 5.1. Proof of Boundedness of Estimation Error Variance Matrix

**Lemma** **2.**  *If* $A,B\in {\mathbb{R}}^{n\times n}$ *satisfy* A>0 *and* B>0 *, and *

(75) $${\left(A+B\right)}^{-1}>A^{-1}-A^{-1}BA^{-1}$$

**Theorem** **2.**  *Assuming that systems (1) and (2) are observable, inequality (74) holds. If the packet dropout rate has a lower bound* $\lambda >1-\frac{1}{\gamma \alpha_{\max}^{2}f_{\max}^{2}}$ *, it can be shown that the error covariance matrix meets the following inequality:*

(76) $$E\left[{\bar{P}}_{k+1}\right]\leq E\left[P_{k+1|k}\right]\leq \bar{p}I$$

**Proof.**  Substitute (18) and (19) into (20), and we can obtain that

(77) $$\begin{array}{l} {\bar{P}}_{k+1}=P_{k+1|k}-\gamma_{k+1}\lambda_{k+1}\left(P_{k+1|k}B_{k+1}^{T}{\left(\begin{array}{l} B_{k+1}P_{k+1|k}B_{k+1}^{T}\\ +R_{k+1} \end{array}\right)}^{-1}\right)B_{k+1}P_{k+1|k}\\ +\left(1-\gamma_{k+1}\right)\times \left[\begin{array}{l} a_{1}P_{k+1|k}-{\left(1+a_{1}\right)}^{2}P_{k+1|k}B_{k+1}^{T}\times \\ {\left[\begin{array}{l} \left(1+a_{1}\right)B_{k+1}P_{k+1|k}B_{k+1}^{T}\\ +\left(1+a_{2}\right)R_{k+1}+\left(1+a_{1}^{-1}+a_{2}^{-1}\right)\rho I \end{array}\right]}^{-1}\\ \times B_{k+1}P_{k+1|k} \end{array}\right] \end{array}$$

□

According to Lemma 2:

(78) $$\begin{array}{l} {\left(B_{k+1}P_{k+1|k}B_{k+1}^{T}+R_{k+1}\right)}^{-1}\\ >B_{k+1}^{-T}P_{k+1}^{-1}B_{k+1}^{-1}-B_{k+1}^{-T}P_{k+1}^{-1}B_{k+1}^{-1}R_{k+1}B_{k+1}^{-T}P_{k+1}^{-1}B_{k+1}^{-1} \end{array}$$

(79) $$\begin{array}{l} \gamma_{k+1}\lambda_{k+1}P_{k+1|k}B_{k+1}{\left(B_{k+1}P_{k+1|k}B_{k+1}^{T}+R_{k+1}\right)}^{-1}B_{k+1}P_{k+1|k}\\ >\gamma_{k+1}\lambda_{k+1}P_{k+1|k}B_{k+1}\\ \times \left(\begin{array}{l} B_{k+1}^{-T}P_{k+1}^{-1}B_{k+1}^{-1}\\ -B_{k+1}^{-T}P_{k+1}^{-1}B_{k+1}^{-1}R_{k+1}B_{k+1}^{-T}P_{k+1}^{-1}B_{k+1}^{-1} \end{array}\right)B_{k+1}P_{k+1|k}\\ =\left(\begin{array}{l} \gamma_{k+1}\lambda_{k+1}B_{k+1}^{-1}\\ -\gamma_{k+1}\lambda_{k+1}B_{k+1}^{-1}R_{k+1}B_{k+1}^{-T}P_{k+1}^{-1}B_{k+1}^{-1} \end{array}\right)B_{k+1}P_{k+1|k}\\ =\gamma_{k+1}\lambda_{k+1}P_{k+1|k}-\gamma_{k+1}\lambda_{k+1}B_{k+1}^{-1}R_{k+1}B_{k+1}^{-T} \end{array}$$

(80) $$\begin{array}{l} {\left[\begin{array}{l} \left(1+a_{1}\right)B_{k+1}P_{k+1|k}B_{k+1}^{T}\\ +\left(1+a_{2}\right)R_{k+1}+\left(1+a_{1}^{-1}+a_{2}^{-1}\right)\rho I \end{array}\right]}^{-1}\\ >\frac{1}{\left(1+a_{1}\right)}B_{k+1}^{-T}P_{k+1}^{-1}B_{k+1}^{-1}-\frac{1}{\left(1+a_{1}\right)}B_{k+1}^{-T}P_{k+1}^{-1}B_{k+1}^{-1}\\ \times \left[\begin{array}{l} \left(1+a_{2}\right)R_{k+1}\\ +\left(1+a_{1}^{-1}+a_{2}^{-1}\right)\rho I \end{array}\right]\frac{1}{\left(1+a_{1}\right)}B_{k+1}^{-T}P_{k+1}^{-1}B_{k+1}^{-1} \end{array}$$

(81) $$\begin{array}{l} {\left(1+a_{1}\right)}^{2}P_{k+1|k}B_{k+1}^{T}\\ \times {\left[\begin{array}{l} \left(1+a_{1}\right)B_{k+1}P_{k+1|k}B_{k+1}^{T}\\ +\left(1+a_{2}\right)R_{k+1}+\left(1+a_{1}^{-1}+a_{2}^{-1}\right)\rho I \end{array}\right]}^{-1}B_{k+1}P_{k+1|k}\\ =\left\{\begin{array}{l} \left(1+a_{1}\right)B_{k+1}^{-1}-\left(1+a_{1}\right)B_{k+1}^{-1}\\ \times \left[\begin{array}{l} \left(1+a_{2}\right)R_{k+1}\\ +\left(1+a_{1}^{-1}+a_{2}^{-1}\right)\rho I \end{array}\right]\\ \times \frac{1}{\left(1+a_{1}\right)}B_{k+1}^{-T}P_{k+1}^{-1}B_{k+1}^{-1} \end{array}\right\}B_{k+1}P_{k+1|k}\\ =\left(1+a_{1}\right)P_{k+1|k}-\left(1+a_{1}\right)B_{k+1}^{-1}\\ \times \left[\begin{array}{l} \left(1+a_{2}\right)R_{k+1}\\ +\left(1+a_{1}^{-1}+a_{2}^{-1}\right)\rho I \end{array}\right]\frac{1}{\left(1+a_{1}\right)}B_{k+1}^{-T} \end{array}$$

So,

(82) $$\begin{array}{l} {\bar{P}}_{k+1}\leq P_{k+1|k}-\gamma_{k+1}\lambda_{k+1}P_{k+1|k}\\ +\gamma_{k+1}\lambda_{k+1}B_{k+1}^{-1}R_{k+1}B_{k+1}^{-T}+\left(1-\gamma_{k+1}\right)\\ \times \left[\begin{array}{l} a_{1}P_{k+1|k}-\left(1+a_{1}\right)P_{k+1|k}\\ +\left(1+a_{1}\right)B_{k+1}^{-1}\left[\begin{array}{l} \left(1+a_{2}\right)R_{k+1}\\ +\left(1+a_{1}^{-1}+a_{2}^{-1}\right)\rho I \end{array}\right]\\ \times \frac{1}{\left(1+a_{1}\right)}B_{k+1}^{-T} \end{array}\right]\\ =P_{k+1|k}-\gamma_{k+1}\lambda_{k+1}\left(P_{k+1|k}-B_{k+1}^{-1}R_{k+1}B_{k+1}^{-T}\right)\\ +\left(1-\gamma_{k+1}\right)\left[\begin{array}{l} B_{k+1}^{-1}\left[\begin{array}{l} \left(1+a_{2}\right)R_{k+1}\\ +\left(1+a_{1}^{-1}+a_{2}^{-1}\right)\rho I \end{array}\right]B_{k+1}^{-T}\\ -P_{k+1|k} \end{array}\right] \end{array}$$

Substitute the above formula into the prediction covariance matrix:

(83) $$\begin{array}{l} P_{k+1|k}=\left(1-\gamma_{k}\lambda_{k}\right)A_{k}{\bar{P}}_{k|k}A_{k}^{T}+Q_{k}+\gamma_{k}\lambda_{k}\left[\left(A_{k}-J_{k}C_{k}\right){\bar{P}}_{k|k}{\left(A_{k}-J_{k}C_{k}\right)}^{T}-S_{k}R_{k}^{-1}S_{k}^{T}\right]\\ \leq \gamma_{k}\left(1-\lambda_{k}\right)A_{k}P_{k|k-1}A_{k}^{T}+Q_{k}-\gamma_{k}\lambda_{k}S_{k}R_{k}^{-1}S_{k}^{T}\\ +\left(1-\gamma_{k}\right)A_{k}B_{k}^{-1}\left[\left(1+a_{2}\right)R_{k}+\left(1+a_{1}^{-1}+a_{2}^{-1}\right)\rho I\right]B_{k}^{-T}A_{k}^{T}\\ +\gamma_{k}\lambda_{k}\left(A_{k}-J_{k}C_{k}\right)B_{k}^{-1}R_{k}B_{k}^{-T}{\left(A_{k}-J_{k}C_{k}\right)}^{T} \end{array}$$

The variance of the state estimation error and the variance of the one-step prediction error are updated separately with new/old measurement data; therefore, $E\left[{\bar{P}}_{k+1}\right]\leq E\left[P_{k+1|k}\right]$. Then, according to (16), taking the upper bound of $P_{k+1}$, we can obtain

(84) $$E\left[P_{k+1|k}\right]\leq E\left[A_{k}{\bar{P}}_{k|k}A_{k}^{T}+Q_{k}+\gamma_{k}\lambda_{k}\left(J_{k}C_{k}{\bar{P}}_{k|k}C_{k}^{T}J_{k}^{T}-J_{k}R_{k}J_{k}^{T}-J_{k}C_{k}{\bar{P}}_{k|k}A_{k}^{T}-A_{k}{\bar{P}}_{k|k}C_{k}^{T}J_{k}^{T}\right)\right]$$

Define $\gamma =E\left[\gamma_{k}\right]$, $\lambda =E\left[\lambda_{k}\right]$ as the average communication rate and arrival probability; then, we have that

(85) $$\begin{array}{l} E\left[P_{k+1|k}\right]\leq \gamma \left(1-\lambda \right)A_{k}E\left[P_{k|k-1}\right]A_{k}^{T}+Q_{k}\\ -\gamma \lambda S_{k}R_{k}^{-1}S_{k}^{T}+\left(1-\gamma \right)A_{k}R_{k}^{-1}\left[\left(1+a_{2}\right)R_{k}+\left(1+a_{1}^{-1}+a_{2}^{-1}\right)\rho I\right]B_{k}^{-T}A_{k}^{T}\\ +\gamma \lambda \left(A_{k}-J_{k}C_{k}\right)B_{k}^{-1}R_{k}B_{k}^{-T}{\left(A_{k}-J_{k}C_{k}\right)}^{T} \end{array}$$

Combine inequality (74):

(86) $$\begin{array}{l} E\left[P_{k+1|k}\right]\leq \gamma \left(1-\lambda \right)\alpha_{\max}^{2}f_{\max}^{2}E\left[P_{k|k-1}\right]\\ +\left\{\begin{array}{l} q+\left(1-\gamma \right)\frac{\alpha_{\max}^{2}f_{\max}^{2}}{\beta_{\min}^{2}h_{\min}^{2}}\left[\begin{array}{l} \left(1+a_{2}\right)r_{\max}\\ +\left(1+a_{1}^{-1}+a_{2}^{-1}\right)\rho \end{array}\right]\\ +\gamma \lambda \frac{{\left(\alpha_{\max}f_{\max}-j_{\min}{\tilde{\alpha }}_{\min}c_{\min}\right)}^{2}r_{\max}}{\beta_{\min}^{2}h_{\min}^{2}}-\gamma \lambda \frac{s_{\min}^{2}}{r_{\max}} \end{array}\right\}I \end{array}$$

Set:

(87) $$p=\max\left\{\begin{array}{l} E\left[P_{1|0}\right],q+\left(1-\gamma \right)\frac{\alpha_{\max}^{2}f_{\max}^{2}}{\beta_{\min}^{2}h_{\min}^{2}}\left[\begin{array}{l} \left(1+a_{2}\right)r_{\max}\\ +\left(1+a_{1}^{-1}+a_{2}^{-1}\right)\rho \end{array}\right]\\ +\gamma \lambda \frac{{\left(\alpha_{\max}f_{\max}-j_{\min}{\tilde{\alpha }}_{\min}c_{\min}\right)}^{2}r_{\max}}{\beta_{\min}^{2}h_{\min}^{2}}-\gamma \lambda \frac{s_{\min}^{2}}{r_{\max}} \end{array}\right\}$$

And (86) is recursively proven:

(88) $$E\left[P_{k+1|k}\right]\leq p\sum_{i=0}^{k}{\left\{\gamma \left(1-\lambda \right)\alpha_{\max}^{2}f_{\max}^{2}\right\}}^{i}I$$

And let $\gamma \left(1-\lambda \right)\alpha_{\max}^{2}f_{\max}^{2}<1$, so that the sum of recursive calculate is satisfied, so that

(89) $$\lambda >1-\frac{1}{\gamma \alpha_{\max}^{2}f_{\max}^{2}}$$

**Remark** **6.**  *From Theorem 2, it can be seen that the critical value for ensuring the boundedness of the estimator is obtained. It can be seen that by adjusting the communication rate* γ *related to the event-triggered threshold* ρ *, the boundedness of the proposed estimator can be guaranteed. At the same time, it has been proven that adjusting threshold* ρ *under bandwidth constraints can achieve a compromise between resource consumption and estimator performance.*

### 5.2. Proof of the Stochastic Stability of Estimation Errors

**Lemma** **3.**  *Assume that there is a stochastic process* $V_{k}\left(\xi_{k}\right)$ *and real number* $v_{\min},v_{\max},\mu >0$ *,* 0<τ≤1 *; then, *

(90) $$v_{\min}{\left\|\xi_{k}\right\|}^{2}\leq V_{k}\left(\xi_{k}\right)\leq v_{\max}{\left\|\xi_{k}\right\|}^{2}$$

(91) $$E\left[V_{k}\left(\xi_{k}\right)|\xi_{k-1}\right]-V_{k-1}\left(\xi_{k-1}\right)\leq \mu -\tau V_{k-1}\left(\xi_{k-1}\right)$$

*and for each Stochastic process,* $\xi_{k}$ *satisfies the above two equations. It can be shown that stochastic process* $\xi_{k}$ *is exponentially bounded in the mean square sense, i.e.,*

(92) $$E\left[{\left\|\xi_{k}\right\|}^{2}\right]\leq \frac{v_{\max}}{v_{\min}}E\left[{\left\|\xi_{0}\right\|}^{2}\right]{\left(1-\tau \right)}^{k}+\frac{\mu }{v_{\min}}\sum_{i=1}^{k-1}{\left(1-\tau \right)}^{i}$$

**Theorem** **3.**  *Consider the nonlinear system (1) and (2) with event-triggered data transmission and packet dropout and assume that inequalities (74) are satisfied. If*

(93) $$0<p_{\min}I\leq {\bar{P}}_{k+1|k+1}\leq P_{k+1|k}\leq p_{\max}I$$

*and for some* ε>0 *,* $E\left[{\left\|{\tilde{x}}_{1|0}\right\|}^{2}\right]\leq \varepsilon$ *, then the prediction error* ${\tilde{x}}_{k+1|k}$ *is bounded in the mean square sense.*

**Proof.**  Define a Lyapunov candidate function:

(94) $$V_{k+1}\left({\tilde{x}}_{k+1|k}\right)={\tilde{x}}_{k+1|k}^{T}P_{k+1|k}^{-1}{\tilde{x}}_{k+1|k}$$

Step 1:

Combining (25) and (28), we can obtain that

(95) $${\tilde{x}}_{k+1|k}=\left(A_{k}-\gamma_{k}\lambda_{k}J_{k}C_{k}\right){\tilde{x}}_{k|k}+w_{k}-\gamma_{k}\lambda_{k}J_{k}v_{k}$$

Prove that the $V_{k+1}\left({\tilde{x}}_{k+1|k}\right)$ conditional expectation of (95) under Gaussian white noise can be obtained:

(96) $$\begin{array}{l} {\left(\alpha_{\min}f_{\min}+\gamma_{k}\lambda_{k}j_{\min}{\overset{⌣}{\alpha }}_{\min}c_{\min}\right)}^{2}p_{\min}I\leq P_{k+1|k}\leq \left(\alpha_{\max}f_{\max}+\gamma_{k}\lambda_{k}j_{\max}{\overset{⌣}{\alpha }}_{\max}c_{\max}\right)p_{\max}I\\ +\left(q_{\max}-\gamma_{k}\lambda_{k}j_{\min}r_{\min}j_{\min}\right)I \end{array}$$

Perform inverse calculation on the above Equation (96), then multiply left by ${\tilde{x}}_{k+1|k}^{T}$ and right by ${\tilde{x}}_{k+1|k}$ to obtain the following:

(97) $$\begin{array}{l} \frac{{\left\|{\tilde{x}}_{k+1|k}\right\|}^{2}}{\left(\alpha_{\max}f_{\max}+\gamma_{k}\lambda_{k}j_{\max}{\overset{⌣}{\alpha }}_{\max}c_{\max}\right)p_{\max}I+\left(q_{\max}-\gamma_{k}\lambda_{k}j_{\min}r_{\min}j_{\min}\right)I}\leq V_{k+1}\left({\tilde{x}}_{k+1|k}\right)\\ \leq \frac{{\left\|{\tilde{x}}_{k+1|k}\right\|}^{2}}{{\left(\alpha_{\min}f_{\min}+\gamma_{k}\lambda_{k}j_{\min}{\overset{⌣}{\alpha }}_{\min}c_{\min}\right)}^{2}p_{\min}I} \end{array}$$

Define $v_{\min}=\frac{{\left\|{\tilde{x}}_{k+1|k}\right\|}^{2}}{\left(\alpha_{\max}f_{\max}+\gamma_{k}\lambda_{k}j_{\max}{\overset{⌣}{\alpha }}_{\max}c_{\max}\right)p_{\max}I+\left(q_{\max}-\gamma_{k}\lambda_{k}j_{\min}r_{\min}j_{\min}\right)I}>0$, $v_{\max}=\frac{{\left\|{\tilde{x}}_{k+1|k}\right\|}^{2}}{{\left(\alpha_{\min}f_{\min}+\gamma_{k}\lambda_{k}j_{\min}{\overset{⌣}{\alpha }}_{\min}c_{\min}\right)}^{2}p_{\min}I}>0$.

This indicates that inequality (90) is valid, and it can also be seen that $V_{k+1}\left({\tilde{x}}_{k+1|k}\right)$ is bounded.

Step 2: Find a real number τ so that  0<τ≤1.

The state estimation error is ${\tilde{x}}_{k+1}=x_{k+1}-{\hat{x}}_{k+1}$, and we combine (17) to obtain

(98) $$\begin{array}{l} {\tilde{x}}_{k+1}=\left(I-\gamma_{k+1}K_{k+1}B_{k+1}-\left(1-\gamma_{k+1}\right)L_{k+1}B_{k+1}\right){\tilde{x}}_{k+1|k}\\ -\left(\gamma_{k+1}K_{k+1}+\left(1-\gamma_{k+1}\right)L_{k+1}\right)\upsilon_{k+1}\\ +\left(1-\gamma_{k+1}\right)L_{k+1}e_{k+1} \end{array}$$

Substituting Equation (98) into Equation (95) yields

(99) $$\begin{array}{l} {\tilde{x}}_{k+1|k}=\left(A_{k}-\gamma_{k}\lambda_{k}J_{k}C_{k}\right)\left(I-\gamma_{k}K_{k}B_{k}-\left(1-\gamma_{k}\right)L_{k}B_{k}\right){\tilde{x}}_{k|k-1}\\ -\left(A_{k}-\gamma_{k}\lambda_{k}J_{k}C_{k}\right)\left(\gamma_{k}K_{k}+\left(1-\gamma_{k}\right)L_{k}\right)\upsilon_{k}\\ +\left(A_{k}-\gamma_{k}\lambda_{k}J_{k}C_{k}\right)\left(1-\gamma_{k}\right)L_{k}e_{k}\\ +w_{k}-\gamma_{k}\lambda_{k}J_{k}v_{k} \end{array}$$

And substituting (99) into $P_{k+1|k}=E\left[{\tilde{x}}_{k+1|k}{\tilde{x}}_{k+1|k}^{T}\right]$, we can obtain that

(100) $$\begin{array}{l} P_{k+1|k}=E\left[{\tilde{x}}_{k+1|k}{\tilde{x}}_{k+1|k}^{T}\right]\\ ={\hat{Q}}_{k}+\left(A_{k}-\gamma_{k}\lambda_{k}J_{k}C_{k}\right)\left(I-\gamma_{k}K_{k}B_{k}-\left(1-\gamma_{k}\right)L_{k}B_{k}\right)P_{k|k-1}\\ \times {\left[\left(A_{k}-\gamma_{k}\lambda_{k}J_{k}C_{k}\right)\left(I-\gamma_{k}K_{k}B_{k}-\left(1-\gamma_{k}\right)L_{k}B_{k}\right)\right]}^{T} \end{array}$$

where

(101) $$\begin{array}{l} {\hat{Q}}_{k}=Q_{k}-\gamma_{k}\lambda_{k}J_{k}R_{k}J_{k}^{T}+\Delta P_{k|k-1}\\ +\left(A_{k}-\gamma_{k}\lambda_{k}J_{k}C_{k}\right)\left(\gamma_{k}K_{k}+\left(1-\gamma_{k}\right)L_{k}\right)R_{k}\\ \times {\left[\left(A_{k}-\gamma_{k}\lambda_{k}J_{k}C_{k}\right)\left(\gamma_{k}K_{k}+\left(1-\gamma_{k}\right)L_{k}\right)\right]}^{T}\\ +\left(A_{k}-\gamma_{k}\lambda_{k}J_{k}C_{k}\right)E\left[Te_{k}N+Ne_{k}T+Ne_{k}N\right]{\left[\left(A_{k}-\gamma_{k}\lambda_{k}J_{k}C_{k}\right)\right]}^{T}\\ -\left(A_{k}-\gamma_{k}\lambda_{k}J_{k}C_{k}\right)\left(\gamma_{k}K_{k}+\left(1-\gamma_{k}\right)L_{k}\right)\left(S_{k}-\gamma_{k}\lambda_{k}R_{k}J_{k}^{T}\right)\\ -{\left[\left(A_{k}-\gamma_{k}\lambda_{k}J_{k}C_{k}\right)\left(\gamma_{k}K_{k}+\left(1-\gamma_{k}\right)L_{k}\right)\left(S_{k}-\gamma_{k}\lambda_{k}R_{k}J_{k}^{T}\right)\right]}^{T}\\ +\left(A_{k}-\gamma_{k}\lambda_{k}J_{k}C_{k}\right)\left(1-\gamma_{k}\right)L_{k}E\left[e_{k}\left(w_{k}-\gamma_{k}\lambda_{k}J_{k}v_{k}\right)\right]\\ +{\left[\left(A_{k}-\gamma_{k}\lambda_{k}J_{k}C_{k}\right)\left(1-\gamma_{k}\right)L_{k}E\left[e_{k}\left(w_{k}-\gamma_{k}\lambda_{k}J_{k}v_{k}\right)\right]\right]}^{T} \end{array}$$

(102) $$\begin{array}{l} T=\left(I-\gamma_{k}K_{k}B_{k}-\left(1-\gamma_{k}\right)L_{k}B_{k}\right){\tilde{x}}_{k|k-1}\\ -\left(\gamma_{k}K_{k}+\left(1-\gamma_{k}\right)L_{k}\right)\upsilon_{k} \end{array}$$

(103) $$N=\left(1-\gamma_{k}\right)L_{k}$$

So, the covariance matrix for errors can be rewritten.

(104) $$\begin{array}{l} P_{k+1|k}=\left[\left(A_{k}-\gamma_{k}\lambda_{k}J_{k}C_{k}\right)\left(I-\gamma_{k}K_{k}B_{k}-\left(1-\gamma_{k}\right)L_{k}B_{k}\right)\right]\\ \times \left\{\begin{array}{l} {\left[\left(A_{k}-\gamma_{k}\lambda_{k}J_{k}C_{k}\right)\left(I-\gamma_{k}K_{k}B_{k}-\left(1-\gamma_{k}\right)L_{k}B_{k}\right)\right]}^{-1}\times {\hat{Q}}_{k}\\ \times {\left[\left(A_{k}-\gamma_{k}\lambda_{k}J_{k}C_{k}\right)\left(I-\gamma_{k}K_{k}B_{k}-\left(1-\gamma_{k}\right)L_{k}B_{k}\right)\right]}^{-T}+P_{k|k-1} \end{array}\right\}\\ \times {\left[\left(A_{k}-\gamma_{k}\lambda_{k}J_{k}C_{k}\right)\left(I-\gamma_{k}K_{k}B_{k}-\left(1-\gamma_{k}\right)L_{k}B_{k}\right)\right]}^{T} \end{array}$$

At the same time, let

(105) $$\begin{array}{l} \Upsilon_{k}={\left[\left(A_{k}-\gamma_{k}\lambda_{k}J_{k}C_{k}\right)\left(I-\gamma_{k}K_{k}B_{k}-\left(1-\gamma_{k}\right)L_{k}B_{k}\right)\right]}^{T}\\ \times {\hat{Q}}_{k}^{-1}\times \left[\left(A_{k}-\gamma_{k}\lambda_{k}J_{k}C_{k}\right)\left(I-\gamma_{k}K_{k}B_{k}-\left(1-\gamma_{k}\right)L_{k}B_{k}\right)\right] \end{array}$$

According to (74), it can be seen that

(106) $$\Upsilon_{k}\leq \frac{{\left[\left(\alpha_{\max}f_{\max}+\gamma_{k}\lambda_{k}j_{\max}{\overset{⌣}{\alpha }}_{\max}c_{\max}\right)\left(\begin{array}{l} 1+\gamma_{k}\beta_{\max}h_{\max}\hat{K}\\ +\left(1-\gamma_{k}\right)\beta_{\max}h_{\max}\hat{L} \end{array}\right)\right]}^{2}}{{\hat{q}}_{\min}}$$

where

(107) $$\left\|K_{k}\right\|\leq \left[p_{\max}\beta_{\max}h_{\max}\right]{\left[\begin{array}{l} {\left(\beta_{\min}h_{\min}\right)}^{2}p_{\min}\\ +\lambda_{k}r_{\min} \end{array}\right]}^{-1}$$

(108) $$\begin{array}{l} \left\|L_{k}\right\|\leq \left[\left(1+a_{1}\right)p_{\max}\beta_{\max}h_{\max}\right]\\ \times {\left[\begin{array}{l} \left(1+a_{1}\right){\left(\beta_{\min}h_{\min}\right)}^{2}p_{\min}+\left(1+a_{2}\right)r_{\min}\\ +\left(1+a_{1}^{-1}+a_{2}^{-1}\right)\rho \end{array}\right]}^{-1} \end{array}$$

Taking the inverse on both sides of (106),

(109) $$\Upsilon_{k}^{-1}\geq \frac{{\hat{q}}_{\min}}{{\left[\left(\alpha_{\max}f_{\max}+\gamma_{k}\lambda_{k}j_{\max}{\overset{⌣}{\alpha }}_{\max}c_{\max}\right)\left(\begin{array}{l} 1+\gamma_{k}\beta_{\max}h_{\max}\hat{K}\\ +\left(1-\gamma_{k}\right)\beta_{\max}h_{\max}\hat{L} \end{array}\right)\right]}^{2}}$$

We substitute (109) into (104), and taking the inverse on both sides of (104), we can obtain

(110) $$\begin{array}{l} {\left[\left(A_{k}-\gamma_{k}\lambda_{k}J_{k}C_{k}\right)\left(I-\gamma_{k}K_{k}B_{k}-\left(1-\gamma_{k}\right)L_{k}B_{k}\right)\right]}^{T}P_{k+1|k}^{-1}\\ \times \left[\left(A_{k}-\gamma_{k}\lambda_{k}J_{k}C_{k}\right)\left(I-\gamma_{k}K_{k}B_{k}-\left(1-\gamma_{k}\right)L_{k}B_{k}\right)\right]\leq \left(1-\tau_{k}\right)P_{k|k-1}^{-1} \end{array}$$

where

(111) $$1-\tau_{k}={\left[1+\frac{{\hat{q}}_{\min}}{{\left[\left(\alpha_{\max}f_{\max}+\gamma_{k}\lambda_{k}j_{\max}{\overset{⌣}{\alpha }}_{\max}c_{\max}\right)\left(\begin{array}{l} 1+\gamma_{k}\beta_{\max}h_{\max}\hat{K}\\ +\left(1-\gamma_{k}\right)\beta_{\max}h_{\max}\hat{L} \end{array}\right)\right]}^{2}p_{\max}}\right]}^{-1}>0$$

Based on the previous inequality assumptions (74),

(112) $$\frac{{\hat{q}}_{\min}}{{\left[\left(\alpha_{\max}f_{\max}+\gamma_{k}\lambda_{k}j_{\max}{\overset{⌣}{\alpha }}_{\max}c_{\max}\right)\left(\begin{array}{l} 1+\gamma_{k}\beta_{\max}h_{\max}\hat{K}\\ +\left(1-\gamma_{k}\right)\beta_{\max}h_{\max}\hat{L} \end{array}\right)\right]}^{2}p_{\max}}>0$$

So, it can be inferred that $0<\tau_{k}<1$.

Step 3: Find a real number $\mu_{k}$ so that $\mu_{k}>0$.

Substitute (99) and (110) into (94), and then, we can obtain the following equation:

(113) $$\begin{array}{l} E\left[V_{k+1}\left({\tilde{x}}_{k+1|k}\right)|{\tilde{x}}_{k+1|k}\right]\\ ={\tilde{x}}_{k|k-1}^{T}\left[P_{k|k-1}^{-1}-\tau_{k}P_{k|k-1}^{-1}\right]{\tilde{x}}_{k|k-1}+\mu_{k} \end{array}$$

Transform the above equation to obtain that

(114) $$\begin{array}{l} E\left[V_{k+1}\left({\tilde{x}}_{k+1|k}\right)|{\tilde{x}}_{k+1|k}\right]-V_{k}\left({\tilde{x}}_{k|k-1}\right)\\ =\mu_{k}-\tau_{k}V_{k}\left({\tilde{x}}_{k|k-1}\right) \end{array}$$

where

(115) $$\mu_{k}=E\left[\begin{array}{l} {\left(\left(A_{k}-\gamma_{k}\lambda_{k}J_{k}C_{k}\right)\left(\gamma_{k}K_{k}+\left(1-\gamma_{k}\right)L_{k}\right)\upsilon_{k}\right)}^{T}P_{k+1|k}^{-1}\left(\left(A_{k}-\gamma_{k}\lambda_{k}J_{k}C_{k}\right)\left(\gamma_{k}K_{k}+\left(1-\gamma_{k}\right)L_{k}\right)\upsilon_{k}\right)\\ +{\left(\left(A_{k}-\gamma_{k}\lambda_{k}J_{k}C_{k}\right)\left(1-\gamma_{k}\right)L_{k}e_{k}\right)}^{T}P_{k+1|k}^{-1}\left(\left(A_{k}-\gamma_{k}\lambda_{k}J_{k}C_{k}\right)\left(1-\gamma_{k}\right)L_{k}e_{k}\right)\\ +w_{k}^{T}P_{k+1|k}^{-1}w_{k}+{\left(\gamma_{k}\lambda_{k}J_{k}v_{k}\right)}^{T}P_{k+1|k}^{-1}\left(\gamma_{k}\lambda_{k}J_{k}v_{k}\right)\\ +{\left(\left(A_{k}-\gamma_{k}\lambda_{k}J_{k}C_{k}\right)\left(I-\gamma_{k}K_{k}B_{k}-\left(1-\gamma_{k}\right)L_{k}B_{k}\right){\tilde{x}}_{k|k-1}\right)}^{T}P_{k+1|k}^{-1}\left(\left(A_{k}-\gamma_{k}\lambda_{k}J_{k}C_{k}\right)\left(1-\gamma_{k}\right)L_{k}e_{k}\right)\\ +{\left(\cdot \right)}^{T}\\ -{\left(\left(A_{k}-\gamma_{k}\lambda_{k}J_{k}C_{k}\right)\left(\gamma_{k}K_{k}+\left(1-\gamma_{k}\right)L_{k}\right)\upsilon_{k}\right)}^{T}P_{k+1|k}^{-1}\left(\left(A_{k}-\gamma_{k}\lambda_{k}J_{k}C_{k}\right)\left(1-\gamma_{k}\right)L_{k}e_{k}\right)+{\left(\cdot \right)}^{T}\\ -{\left(\left(A_{k}-\gamma_{k}\lambda_{k}J_{k}C_{k}\right)\left(\gamma_{k}K_{k}+\left(1-\gamma_{k}\right)L_{k}\right)\upsilon_{k}\right)}^{T}P_{k+1|k}^{-1}\left(w_{k}-\gamma_{k}\lambda_{k}J_{k}v_{k}\right)+{\left(\cdot \right)}^{T}\\ +{\left(\left(A_{k}-\gamma_{k}\lambda_{k}J_{k}C_{k}\right)\left(1-\gamma_{k}\right)L_{k}e_{k}\right)}^{T}P_{k+1|k}^{-1}\left(w_{k}-\gamma_{k}\lambda_{k}J_{k}v_{k}\right)+{\left(\cdot \right)}^{T} \end{array}\right]$$

The above equation is a scalar, so when taking the trace of the matrix for the above equation, it does not change its own value.

Combining Lemma 1, it can be obtained that

(116) $$\mu_{k}\leq tr\left\{\begin{array}{l} {\left(\left(A_{k}-\gamma_{k}\lambda_{k}J_{k}C_{k}\right)\left(\gamma_{k}K_{k}+\left(1-\gamma_{k}\right)L_{k}\right)\right)}^{T}P_{k+1|k}^{-1}\left(\left(A_{k}-\gamma_{k}\lambda_{k}J_{k}C_{k}\right)\left(\gamma_{k}K_{k}+\left(1-\gamma_{k}\right)L_{k}\right)\right)R_{k}\\ +{\left(\left(A_{k}-\gamma_{k}\lambda_{k}J_{k}C_{k}\right)\left(1-\gamma_{k}\right)L_{k}\right)}^{T}P_{k+1|k}^{-1}\left(\left(A_{k}-\gamma_{k}\lambda_{k}J_{k}C_{k}\right)\left(1-\gamma_{k}\right)L_{k}\right)\rho I\\ +P_{k+1|k}^{-1}Q_{k}+\gamma_{k}\lambda_{k}J_{k}P_{k+1|k}^{-1}J_{k}^{T}R_{k}\\ +a_{3}{\left[{\left(\left(A_{k}-\gamma_{k}\lambda_{k}J_{k}C_{k}\right)\left(\begin{array}{l} I-\gamma_{k}K_{k}B_{k}\\ -\left(1-\gamma_{k}\right)L_{k}B_{k} \end{array}\right)\right)}^{T}P_{k+1|k}^{-1}\right]}^{T}P_{k|k-1}\\ \times \left[{\left(\left(A_{k}-\gamma_{k}\lambda_{k}J_{k}C_{k}\right)\left(I-\gamma_{k}K_{k}B_{k}-\left(1-\gamma_{k}\right)L_{k}B_{k}\right)\right)}^{T}P_{k+1|k}^{-1}\right]\\ +a_{4}{\left[{\left(\left(A_{k}-\gamma_{k}\lambda_{k}J_{k}C_{k}\right)\left(\gamma_{k}K_{k}+\left(1-\gamma_{k}\right)L_{k}\right)\right)}^{T}P_{k+1|k}^{-1}\right]}^{T}R_{k}\\ \times \left[{\left(\left(A_{k}-\gamma_{k}\lambda_{k}J_{k}C_{k}\right)\left(\gamma_{k}K_{k}+\left(1-\gamma_{k}\right)L_{k}\right)\right)}^{T}P_{k+1|k}^{-1}\right]\\ +a_{5}{\left[{\left(\left(A_{k}-\gamma_{k}\lambda_{k}J_{k}C_{k}\right)\left(\gamma_{k}K_{k}+\left(1-\gamma_{k}\right)L_{k}\right)\right)}^{T}P_{k+1|k}^{-1}\right]}^{T}R_{k}\\ \times \left[{\left(\left(A_{k}-\gamma_{k}\lambda_{k}J_{k}C_{k}\right)\left(\gamma_{k}K_{k}+\left(1-\gamma_{k}\right)L_{k}\right)\right)}^{T}P_{k+1|k}^{-1}\right]\\ +a_{6}{\left(\left(Q_{k}-\gamma_{k}\lambda_{k}J_{k}R_{k}\right)P_{k+1|k}^{-1}\right)}^{T}\left(\left(Q_{k}-\gamma_{k}\lambda_{k}J_{k}R_{k}\right)P_{k+1|k}^{-1}\right)\\ +\left(a_{3}^{-1}+a_{4}^{-1}+a_{6}^{-1}\right)\left(\left(A_{k}-\gamma_{k}\lambda_{k}J_{k}C_{k}\right)\left(1-\gamma_{k}\right)L_{k}\right)\rho I{\left(\left(A_{k}-\gamma_{k}\lambda_{k}J_{k}C_{k}\right)\left(1-\gamma_{k}\right)L_{k}\right)}^{T}\\ +a_{5}^{-1}\left(Q_{k}-\gamma_{k}\lambda_{k}J_{k}R_{k}\right)\left(Q_{k}-\gamma_{k}\lambda_{k}J_{k}R_{k}\right) \end{array}\right\}$$

In the above Equation (116), $a_{3}$, $a_{4}$, $a_{5}$, and $a_{6}$ are positive numbers, and it can be seen that $\mu_{k}$ is a positive number, and it has an upper bound $\mu_{\max}$.

Step 4: Prove that the stochastic process ${\tilde{x}}_{k+1|k}$ is exponentially bounded in mean square.

From (113)–(116), it can be obtained that

(117) $$E\left[V_{k+1}\left({\tilde{x}}_{k+1|k}\right)|{\tilde{x}}_{k+1|k}\right]-V_{k}\left({\tilde{x}}_{k|k-1}\right)\leq \mu_{k}-\tau_{k}V_{k}\left({\tilde{x}}_{k|k-1}\right)$$

The above equation is the second inequality of Lemma 3. From (97)–(117), we can obtain that the stochastic process ${\tilde{x}}_{k+1|k}$ is bounded in the mean square sense.

Step 5: Prove that the estimation error is bounded in the mean square.

From (28), the estimation error at time k can be written as

(118) $${\tilde{x}}_{k|k}={\left(A_{k}-\gamma_{k}\lambda_{k}J_{k}C_{k}\right)}^{-1}\left[{\tilde{x}}_{k+1|k}-\left(w_{k}-\gamma_{k}\lambda_{k}J_{k}v_{k}\right)\right]$$

The mean square error expression for the estimation error is as follows:

(119) $$\begin{array}{l} E\left[\left\|{\tilde{x}}_{k|k}\right\|\right]={\left(A_{k}-\gamma_{k}\lambda_{k}J_{k}C_{k}\right)}^{-1}E\left[\left\|{\tilde{x}}_{k+1|k}\right\|\right]{\left(A_{k}-\gamma_{k}\lambda_{k}J_{k}C_{k}\right)}^{-T}\\ +{\left(A_{k}-\gamma_{k}\lambda_{k}J_{k}C_{k}\right)}^{-1}E\left[\left\|w_{k}\right\|\right]{\left(A_{k}-\gamma_{k}\lambda_{k}J_{k}C_{k}\right)}^{-T}\\ ={\left(\alpha_{k}F_{k}-\gamma_{k}\lambda_{k}J_{k}C_{k}\right)}^{-1}E\left[\left\|{\tilde{x}}_{k+1|k}\right\|\right]{\left(\alpha_{k}F_{k}-\gamma_{k}\lambda_{k}J_{k}C_{k}\right)}^{-T}\\ +{\left(\alpha_{k}F_{k}-\gamma_{k}\lambda_{k}J_{k}C_{k}\right)}^{-1}E\left[\left\|w_{k}\right\|\right]{\left(\alpha_{k}F_{k}-\gamma_{k}\lambda_{k}J_{k}C_{k}\right)}^{-T} \end{array}$$

Combining (74), the mean square error of the estimation is

(120) $$E\left[\left\|{\tilde{x}}_{k|k}\right\|\right]\leq {\left(\alpha_{k}F_{k}-\gamma_{k}\lambda_{k}J_{k}C_{k}\right)}^{-2}E\left[\left\|{\tilde{x}}_{k+1|k}\right\|+\left\|w_{k}\right\|\right]$$

Similar to the proof that stochastic process ${\tilde{x}}_{k+1|k}$ is mean square-bounded, it can also be proven that $w_{k}$ is mean square-bounded. Therefore, it can be proven that the estimation error ${\tilde{x}}_{k|k}$ is military-bounded. □

## 6. Numerical Example

In this section, we use a simulation example to demonstrate the effectiveness of the algorithm that is proposed in this paper. Consider the following passive tracking model:

(121) $$x_{k+1}=\left[\begin{array}{ccccc} 1 & \frac{\sin\left(\Omega_{k}T\right)}{\Omega_{k}} & 0 & \frac{\cos\left(\Omega_{k}T\right)-1}{\Omega_{k}} & 0\\ 0 & \cos\left(\Omega_{k}T\right) & 0 & -\sin\left(\Omega_{k}T\right) & 0\\ 0 & \frac{1-\cos\left(\Omega_{k}T\right)}{\Omega_{k}} & 1 & \frac{\sin\left(\Omega_{k}T\right)}{\Omega_{k}} & 0\\ 0 & \sin\left(\Omega_{k}T\right) & 0 & \cos\left(\Omega_{k}T\right) & 0\\ 0 & 0 & 0 & 0 & 1 \end{array}\right]x_{k}+\Gamma_{k}\omega_{k}$$

(122) $$y_{k}=\left[\begin{array}{c} \sqrt{x_{1,k+1}^{2}+x_{3,k+1}^{2}}\\ \arctan\left(x_{3,k+1}/x_{1,k+1}\right) \end{array}\right]+\Psi_{k}\upsilon_{k}$$

where $\Gamma_{k}={\left[\begin{array}{ccccc} 10 & 1 & 10 & 1 & 10^{-4} \end{array}\right]}^{T}$, $\Psi_{k}={\left[\begin{array}{cc} 100 & 10^{-5} \end{array}\right]}^{T}$, the initial true state value is $x_{0}={\left[\begin{array}{ccccc} 1000\;m & 300\;m/s & 1000\;m & 0\;m/s & 0.052\;\mathrm{rad} \end{array}\right]}^{T}$, the initial estimated state values is ${\hat{x}}_{0}={\left[\begin{array}{ccccc} 1000\;m & 300\;m/s & 1000\;m & 0\;m/s & 0.052\;\mathrm{rad} \end{array}\right]}^{T}$, and the initial estimated state variance is ${\hat{P}}_{0}=diag\left[\begin{array}{ccccc} 100\;m^{2} & 10\;m^{2}/s^{2} & 100\;m^{2} & 10\;m^{2}/s^{2} & 10^{-4}{\mathrm{rad}}^{2}/s^{2} \end{array}\right]$. State that vector $x_{k}={\left[x,v_{x},y,v_{y},\theta \right]}^{T}$; the components are position and velocity along the x-axis, position and velocity along the y-axis, and turn rate. The process noise $\omega_{k}∼N\left(0,Q_{k}\right)$ and the measurement noise $v_{k}∼N\left(0,R_{k}\right)$ are both Gaussian white noises with zero means. Set $Q_{k}=10$, $R_{k}=1$, and $S_{k}=1$ and the event-triggered thresholds to 500,000 and 2,000,000, respectively. The values of $a_{1}$, $a_{2}$ are considered to be 0.5.

The state estimation error (Error) and root mean square error (RMSE) for 120 Monte Carlo runs are calculated as follows:

(123) $$Error=\frac{1}{N}\sum_{n=1}^{N}\left(x_{k}^{\left(n\right)}-{\hat{x}}_{k|k}^{\left(n\right)}\right),1\leq k\leq 120$$

(124) $$RMSE=\sqrt{\frac{1}{N}\sum_{n=1}^{N}{\left(x_{k}^{\left(n\right)}-{\hat{x}}_{k|k}^{\left(n\right)}\right)}^{2}},1\leq k\leq 120$$

where $x_{k}^{\left(n\right)}$ and ${\hat{x}}_{k|k}^{\left(n\right)}$ denote the true state and the estimate of the kth step in each Monte Carlo run, respectively. And N = 120 denotes the number of Monte Carlo runs.

PDR represents the packet dropout rate, and ρ represents the event-triggered threshold. The performance of the proposed algorithm is analyzed by comparing the effect of state estimation under different packet dropout rates and different event-triggered thresholds. The simulation results show that by properly adjusting the threshold of the event-triggered mechanism, the expected estimation quality can be obtained while significantly reducing the communication rate.

**Figure 1:**
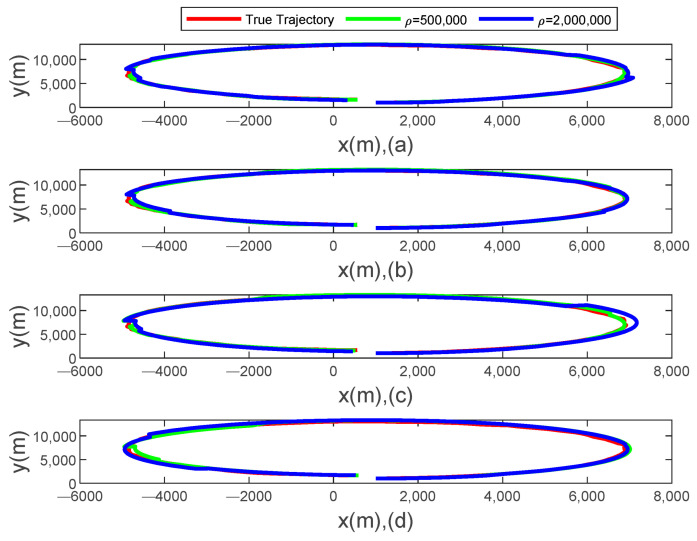
Tracking results of the proposed algorithm. (**a**) PDR = 0.1. (**b**) PDR = 0.3. (**c**) PDR = 0.5. (**d**) PDR = 0.7.

Figure 1 offers a visual representation of the position tracking outcomes that are obtained through the algorithm presented in this study. As the packet dropout rate varies, the algorithm demonstrates consistent performance, even under challenging conditions. When the packet dropout rate remains constant, the position estimation maintains stability as the event-triggered threshold is adjusted. This adaptability is crucial in real-world applications, where environmental factors and network conditions constantly change. Furthermore, with a consistent event-triggered threshold, the tracking remains robust despite changes in the packet dropout rate. This remarkable consistency highlights the efficacy and numerical stability of the algorithm, making it a suitable candidate for a wide range of applications, including target tracking, robotics, and networked systems. So, the filter design of the nonlinear system with related noise and packet dropout based on an event-triggered mechanism proposed in this paper can be applied to the latest controller design of quadcopter drones and offshore container cranes. It can provide control object position, speed, and attitude information for the controller, while it can also reduce the communication energy loss in the system [32,33].

**Figure 2:**
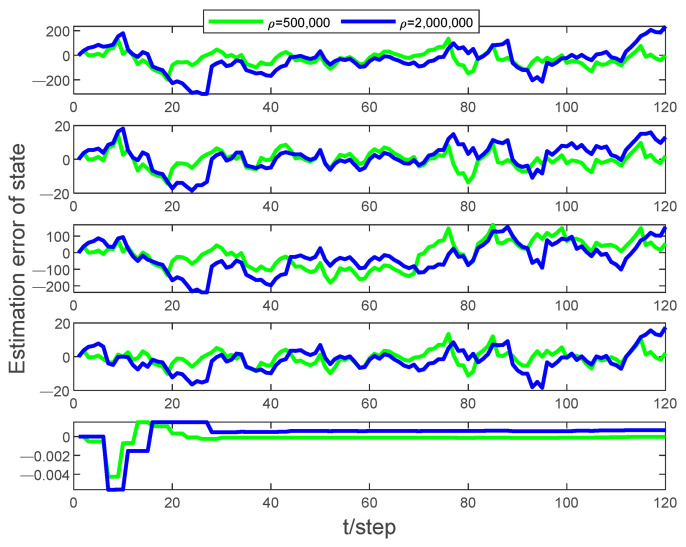
Estimation error of the state $\left(x,v_{x},y,v_{y},\theta \right)$ at different event-triggered thresholds with PDR = 0.1.

Figure 2 presents a graphic representation of the state estimation error when the event-triggered thresholds are set to $5\times 10^{5}$ and $2\times 10^{6}$, respectively, with a packet dropout rate of PDR=0.1. As the event-triggered threshold increases, the state estimation error also increases, but it remains within manageable bounds. This observation highlights the robustness of the algorithm in handling varying event-triggered thresholds, even under challenging network conditions.

**Figure 3:**
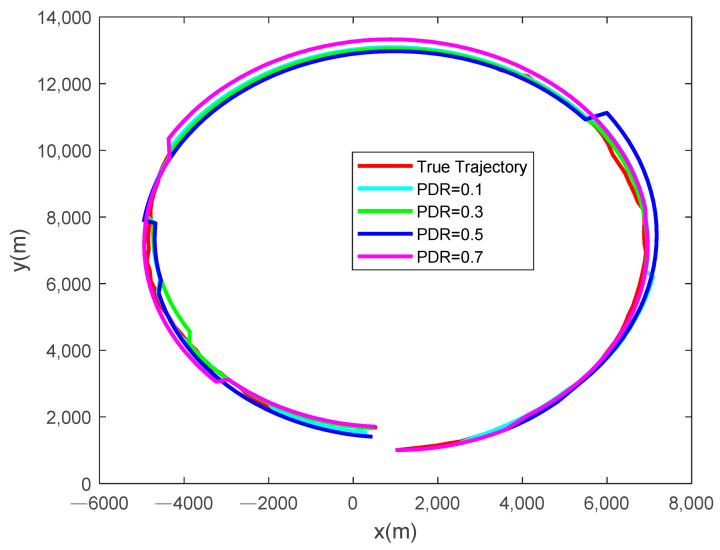
Tracking results at different packet dropout rates with $\rho =5\times 10^{5}$.

**Figure 4:**
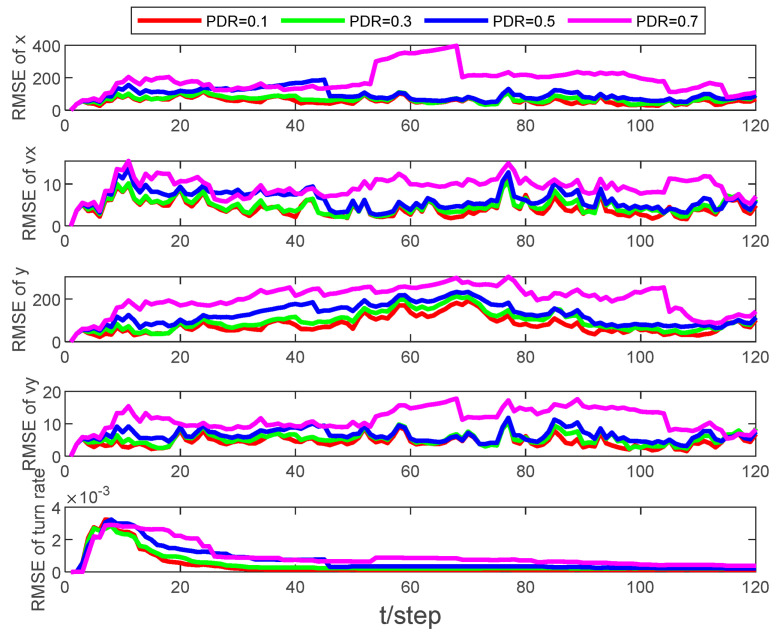
RMSE of state estimation at different packet dropout rates with $\rho =5\times 10^{5}$.

Figure 3 and Figure 4 present the tracking results and the root mean square error (RMSE) of the state estimation, respectively, at different packet dropout rates with an event-triggered threshold set to $\rho =5\times 10^{5}$. As the packet dropout rate increases, the quality of the estimation degrades, yet the tracking RMSE remains stable and within manageable bounds.

**Figure 5:**
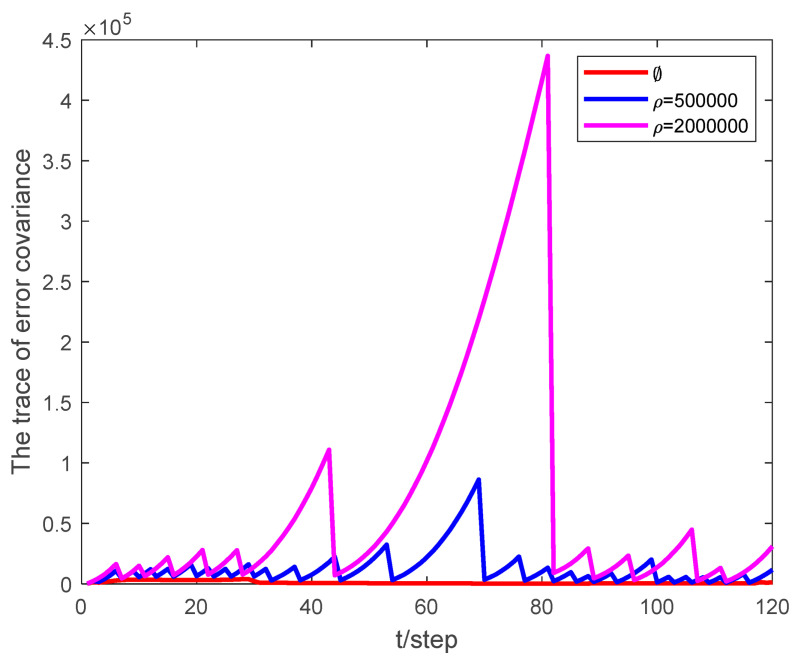
The trace of state error covariance at different event-triggered thresholds with PDR = 0.1.

**Figure 6:**
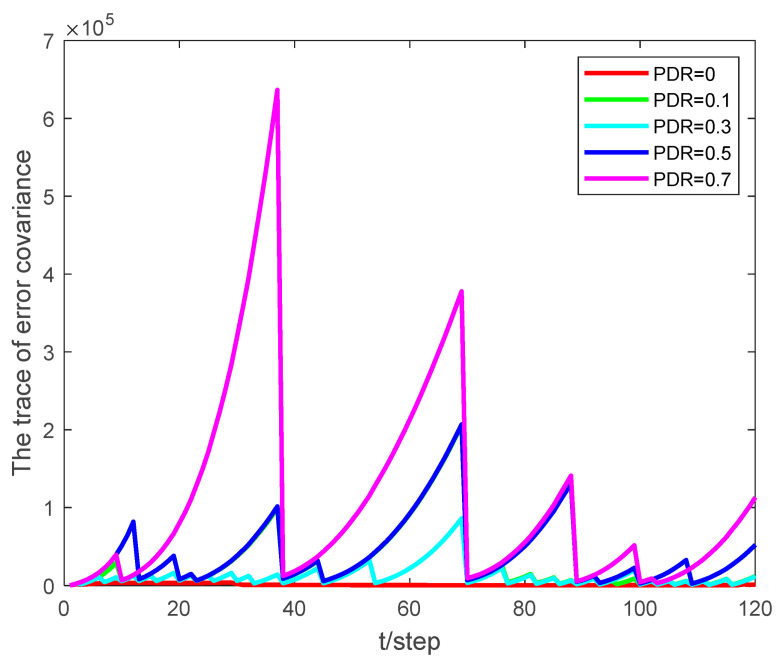
The trace of state error covariance at different packet dropout rates with $\rho =5\times 10^{5}$.

Examining Figure 5 and Figure 6, it becomes evident that as the packet dropout rate or event-triggered threshold increases, the algorithm’s performance degrades. This finding underscores the importance of striking an optimal balance between the transmission rate and estimator performance. By adjusting the event-triggered threshold appropriately, it is possible to achieve this balance, ensuring reliable tracking under various network conditions. This adaptability is crucial in real-world applications, where environmental factors and network conditions constantly change.

## 7. Conclusions

In this paper, the problem of event-triggered state estimation for nonlinear systems with packet dropout and correlated noise is studied, and a nonlinear filtering algorithm based on a CKF is proposed. Research has shown that event-triggered transmission mechanisms can reduce the transmission rate of measurement data in network systems and can achieve a compromise between the communication transmission rate and filter estimation performance by adjusting the event-triggered threshold appropriately. Then, the numerical implementation steps based on the third-degree spherical–radial cubature rule are given, the stochastic stability of the designed filter is analyzed, and the lower bound of the packet dropout rate of the measurement data related to the event-triggering threshold is obtained, ensuring the convergence of the designed filter. Finally, the effectiveness of the proposed filter is verified through simulation numerical examples of target tracking.

## Data Availability

Data are contained within the article.

## References

[1] Zhang H., Zhou X., Wang Z., Yan H., Sun J. (2019). Adaptive consensus based distributed target tracking with dynamic cluster in sensor networks. IEEE Trans. Cybern..

[2] Liu B., Li Z., Chen X., Huang Y., Liu X. (2018). Recognition and vulnerability analysis of key nodes in power grid based on complex network centrality. IEEE Trans. Circuits Syst. II Express Briefs.

[3] Ho Y.C., Cassandras C. (1983). A new approach to the analysis of discrete event dynamic system. Automatica.

[4] Aströmand K.J., Bo B. (1999). Comparison of periodic and event based sampling for first order stochastic systems. Proc. IFAC World Congr..

[5] Miskowicz M. (2006). Send-on-delta concept: An event-based data reporting strategy. Sensors.

[6] Suh Y. (2007). Send-on-delta sensor data transmission with a linear predictor. Sensors.

[7] Shi D., Chen T., Shi L. (2014). Event-triggered maximum likelihood state estimation. Automatica.

[8] Trimpe S., D’Andrea R. (2014). Event-based state estimation with variance-based triggering. IEEE Trans Autom. Control.

[9] Wu J., Jia Q.S., Johansson K.H., Shi L. (2013). Event-based sensor data scheduling: Trade-off between communication rate and estimation quality. IEEE Trans. Autom. Control.

[10] Han D., Mo Y., Wu J., Weerakkody S., Sinopoli B., Shi L. (2015). Stochastic event-triggered sensor schedule for remote state estimation. IEEE Trans. Autom. Control.

[11] Bar-Shalom Y., Li X.R., Kirubarajan T. (2004). Estimation with Applications to Tracking and Navigation: Theory Algorithms and Software.

[12] Nørgaard M., Poulsen N.K., Ravn O. (2000). New developments in state estimation for nonlinear system. Automatica.

[13] Julier S.J. (2004). Unscented filtering and nonlinear estimation. Proc. IEEE.

[14] Arasaratnam I., Haykin S. (2009). Cubature Kalman Filters. IEEE Trans. Autom. Control.

[15] Doucet A., Godsill S., Andrieu C. (2000). On sequential Monte Carlo sampling methods for Bayesian filtering. Stat. Comput..

[16] Zhao K., Li P., Song S.-M. (2018). Gaussian Filter for Nonlinear Stochastic Uncertain Systems with Correlated Noises. IEEE Sens. J..

[17] Zhao K., Tan L.G., Song S.M. (2019). Gaussian Filter for Nonlinear Networked Systems with Synchronously Correlated Noises and One-Step Randomly Delayed Measurements and Multiple Packet Dropouts. IEEE Sens. J..

[18] Tan L.G., Xu C., Wang Y.F., Wei H.N., Zhao K., Song S.M. (2020). Gaussian recursive filter for nonlinear systems with finite-step correlated noises and packet dropout compensations. Meas. Sci. Rev..

[19] Lin H.L., Sun S.L. (2019). Globally optimal sequential and distributed fusion state estimation for multi-sensor system with cross-correlated noises. Automatica.

[20] Wang N., Sun S.L. (2021). Event-triggered sequential fusion filters based on estimators of observation noise for multi-sensor systems with correlated noises. Digit. Signal Process..

[21] Cheng G.R., Ma M.C., Tan L.G., Song S.M. (2022). Event-triggered sequential fusion filter for nonlinear multi-sensor systems with correlated noise based on observation noise estimation. IEEE Sens. J..

[22] Wang G., Chen J., Sun J. (2013). Stochastic stability of extended filtering for non-linear systems with measurement packet losses. IET Control Theory.

[23] Liu X., Li L., Li Z., Iu H.H., Fernando T. (2017). Stochastic stability of modified extended Kalman filter over fading channels with transmission failure and signal fluctuation. Signal Process..

[24] Li L., Yu D., Xia Y., Yang H. (2019). Remote nonlinear state estimation with stochastic event-triggered sensor schedule. IEEE Trans. Cybern..

[25] Li L., Yu D., Yang H., Yan C. UKF for nonlinear systems with event-triggered data transmission and packet dropout. Proceedings of the 2016 3rd International Conference on Informative and Cybernetics for Computational Social Systems (ICCSS).

[26] Li S. (2021). Application of event-triggered cubature Kalman filter for remote nonlinear state estimation in wireless sensor network. IEEE Trans. Ind. Electron..

[27] Wanasinghe T.R., Mann G.K.I., Gosine R.G. Stability Analysis of the Discrete-Time Cubature Kalman Filter. Proceedings of the 2015 54th IEEE Conference on Decision and Control (CDC).

[28] Sinopoli B., Schenato L., Franceschetti M., Poolla K., Jordan M.I., Sastry S. (2004). Kalman filtering with intermittent observations. IEEE Trans Autom. Control.

[29] Kooshkbaghi M., Marquez H.J. (2020). Event-triggered Discrete-Time Cubature Kalman Filter for Nonlinear Dynamical Systems with Packet Dropout. IEEE Trans. Autom. Control.

[30] Shi D., Chen T., Shi L. (2014). An event-triggered approach to state estimation with multiple point and set-valued measurements. Automatica.

[31] Liu X., Li L., Li Z., Fernando T., Iu H.H. (2017). Stochastic stability condition for the extended Kalman filter with intermittent observations. IEEE Trans. Circuits Syst. II Express Briefs.

[32] Pouzesh M., Mobayen S. (2022). Event-triggered fractional-order sliding mode control technique for stabilization of distributed quadrotor unmanned aerial vehicles. Aerosp. Sci. Technol..

[33] Zanjani M.S., Mobayen S. (2023). Event-triggered global sliding mode controller design for anti-sway control of offshore container cranes. Ocean. Eng..

